# Reduction of alternative polarization of macrophages by short-term activated hepatic stellate cell-derived small extracellular vesicles

**DOI:** 10.1186/s13046-025-03380-0

**Published:** 2025-04-10

**Authors:** Yufeng Sun, Min Zhao, Li Cheng, Xiaoqian He, Shiqi Shen, Jiaying Lv, Junyu Zhang, Qian Shao, Wenxuan Yin, Fengbo Zhao, Rui Sun, Peng Lu, Yuhua Ji, Xin Wei Wang, Juling Ji

**Affiliations:** 1 Department of Pathology, Medical School of Nantong University, Nantong, 226001 China; 2 Key Laboratory of Microenvironment and Translational Cancer Research, Nantong, 226001 China; 3 Key Laboratory of Neuroregeneration of Jiangsu and Ministry of Education, Nantong University, Nantong, 226001 China; 4 Basic Medical Research Center, Medical School of Nantong University, Nantong, 226001 China; 5 Department of Immunology and Microbiology, College of Life Science and Technology, Jinan University, Guangzhou, 510632 China; 6 Laboratory of Human Carcinogenesis, Center for Cancer Research, National Cancer Institute, Bethesda, MD 20892 USA; 7 Liver Cancer Program, Center for Cancer Research, National Cancer Institute, Bethesda, MD, 20892 USA; 8 Department of Pathology, Affiliated Hospital of Nantong University, Nantong, 226001 China

## Abstract

**Background:**

Activated hepatic stellate cells (HSCs) induce alternative (M2) polarization of macrophages and contribute to the progression of fibrosis and hepatocellular carcinoma (HCC). However, the effects of small extracellular vesicles released by HSCs (HSC-sEVs) during activation remain largely unknown.

**Methods:**

The aim of this study was to investigate the role of extracellular vesicles released by HSCs (HSC-sEVs) at different stages of activation in macrophage polarization. The effects of sEVs from short-term activated and long-term activated HSCs on liver macrophages was studied. Small RNA sequencing analyses were performed to obtain differential miRNAs transported by the short-term and long-term activated HSC- sEVs. The in vivo effects of short-term activated HSC-sEV-specific miRNA on liver macrophage and liver fibrosis were confirmed in a CCl4-induced liver injury mouse model. To study the tumor suppressive effects of the macrophages educated by short-term activated HSC-sEV-specific miRNA, human hepatoma cells were mixed and subcutaneously cotransplanted with miR-99a-5p mimic-pretreated macrophages.

**Results:**

We found that consistent with activated HSCs, long-term activated HSC-sEVs (14dHSC-sEVs) induce bone marrow-derived monocytes (MOs) toward an M2 phenotype, but short-term activated HSC-sEVs (3dHSC-sEVs) induce the resident macrophages (Kupffer cells, KCs) toward a classically activated (M1) phenotype. We identified five 3dHSC-sEV-specific miRNAs, including miR-99a-5p. In vitro and in vivo experiments support that miR-99a-5p negatively regulates alternative polarization of macrophages, decreases collagen deposition in chronic liver injury model, and suppresses the progression of hepatoma in a xenograft model partially by targeting CD93.

**Conclusion:**

Collectively, our work reveals an unexpected proinflammatory role of 3dHSC-sEVs, preliminarily explores the underlying mechanism, and evaluates the therapeutic potential of 3dHSC-sEV-specific miR-99a-5p for liver fibrosis and tumorigenesis.

**Supplementary Information:**

The online version contains supplementary material available at 10.1186/s13046-025-03380-0.

## Introduction

Liver cancer is the second most common cause of cancer-related death worldwide, with increasing incidence and mortality rates [1]. Hepatocellular carcinoma (HCC) alone accounts for 90% of all cases of primary liver cancer. Approximately 80–90% of HCCs develop from liver fibrosis or cirrhosis [2]. The ratio of HCC incidence for the cirrhotic state was 8.73-fold of non-cirrhotic state [3].

Hepatic stellate cells (HSCs) play central roles in liver pathogenesis, including cirrhosis and hepatocellular carcinoma [4, 5]. They are pericytes in perisinusoidal Disse space and represent approximately 15% of total resident cells in the normal human liver [6]. Quiescent HSCs store up to 80% of the total body vitamin A, maintain the balance of the liver extracellular matrix, produce a variety of cytokines, and regulate the diameter of the liver blood sinus. Upon liver injury caused by various factors, including hepatitis virus, alcohol, drugs, and immune or metabolic disorders, HSCs are activated and transdifferentiated into myofibroblasts that produce more ECM components and are critical contributors to liver fibrosis and cirrhosis [7]. It has been reported that an activated HSC-associated cirrhotic microenvironment is a prognostic factor for HCC independent of etiology and tumor cell biological characteristics [8, 9]. A comprehensive understanding of the role of HSCs in intrahepatic immune regulation will lead to promising strategies for the early prevention and treatment of liver cancer [10].

Small extracellular vesicles (sEVs) are a group of nanovesicles with a diameter generally smaller than 200 nm and mainly derived from the endosomal system, coated with a lipid bilayer and containing proteins, lipids, and nucleic acid cargoes [11]. The sEV cargo represents a snapshot of the parental cell at the time of release and can change depending on the status of the cell [12]. Thus, sEVs are an essential method of information transmission between cells [13, 14]. Considering the limited space and restricted direct contact among cells in the sinusoids, but the strong impact of activated HSCs on HCC progression [8], we speculated that sEVs produced by HSCs might contain unique signaling molecules to communicate with immune cells and potentially malignant cells and contribute to cirrhosis-related liver malignancy.

MicroRNAs (miRNAs) are essential components of sEVs [15]. They are small noncoding RNAs involved in post-transcriptional gene regulation and contribute to establishing gene expression patterns in most cell types [16]. With the intake of sEVs, sEV-miRNAs enter and modulate the biofunction of recipient cells [17]. Given the substantial phenotypic changes in HSCs during their activation and the remarkable differences in gene expression between quiescent and activated HSCs [18, 19], we proposed that the miRNA composition carried by sEVs released during different stages of HSC activation could be very different, and these sEVs loaded with different miRNAs might act differently in the microenvironment.

We previously reported that long-term activated HSCs favored M2 polarization of macrophages and accelerated the progression of HCC in chronically injured fibrotic liver [8]. It is largely unknown how short-term activated HSCs during the early stage of liver injury affect the differentiation of macrophage and the progression of liver malignancy. In the present study, we compared the effects of short-term and long-term culture-activated HSCs on macrophage differentiation. The potential mechanism was explored, focusing on the differential miRNAs delivered by corresponding HSC-sEVs. An encouraging finding is that contrary to the immune tolerance effect of long-term activated HSC-sEVs, short-term activated HSC-sEVs could induce proinflammatory activation of macrophages. We then identified the sEV-miRNAs altered during HSCs activation and validated the critical role of a key miRNA.

Our study supports that HSCs at different stages of activation play different roles in macrophage polarization and provides a new perspective for the study of liver fibrosis and tumor-related immune microenvironments.

## Materials and methods

### Animals and cell lines

Male Sprague-Dawley rats (body weight 450–550 g), 8-week-old male C57BL/6J mice, and 5-week-old male BALB/c nude mice were purchased from Shanghai SLAC Laboratory Animal Co, Ltd and housed in the animal facility of Nantong University. All animal experimental protocols were reviewed and approved by the Animal Ethics Committee of Nantong University.

The human HSC line LX2 (a gift from S. Friedman, Mount Sinai School of Medicine, New York, USA) was maintained in DMEM (Gibco, Carlsbad, CA, USA) containing 2% FBS (Gibco). The human liver cell line LO2, hepatoma HepG2, Huh1 and Huh7 cell lines, human embryonic kidney HEK 293T cell line and human monocyte-like THP-1 cell line were obtained from the Cell Bank of the Chinese Academy of Sciences. The HepG2, Huh7 and HEK 293T cell lines were maintained in DMEM containing 10% FBS (Gibco). The THP-1 cell line was maintained in RPMI medium 1640 (Gibco) containing 10% FBS (Gibco) and 0.05 mM β-mercaptoethanol (Invitrogen, Carlsbad, CA, USA). All cells were kept in a humidified incubator at 37 °C with 5% CO_2_. In the present study, all THP-1 cells used were pretreated with 10 ng/mL phorbol-12-myristate-13-acetate (PMA) for 48 h to obtain THP-1-derived macrophages [20]. The term THP-1 in the present study refers to THP-1-derived macrophages.

### Primary rat HSC, liver resident macrophage (Kupffer cell, KC) and bone marrow-derived monocyte (MO) isolation, purification and identification

The nonparenchymal rat liver cells were prepared using a two-step enzymatic digestion method. HSC-enriched cells were obtained from these cells by density gradient centrifugation as described previously [19] and further purified by magnetic-activated cell sorting (MACS) negative selection (Miltenyi Biotec GmbH, Bergisch Gladbach, Germany) to remove possible contamination of CD45 + myeloid cells and CD31 + endothelial cells according to the manufacturer’s instructions. As shown by the intracellular autofluorescence of vitamin A and trypan blue exclusive assay, the purity and vitality of the purified HSCs were both above 98%. HSCs were seeded in 25 cm^2^ culture flasks or onto coverslips and cultured in DMEM (Gibco) with 10% Exo-free FBS (System Biosciences, Inc., Mountain View, CA, USA) at 37 °C in a 5% CO2 atmosphere incubator. Lipid droplets were stained with Lipid Droplet Green Fluorescent Detection Kit BODIPY 493/503 (Beyotime Biotechnology, Shanghai, China), according to manufacturer’s instructions*.* The culture medium was refreshed every 3 days. Day 0, Day 3, Day 7 and Day 14 HSCs were collected and lysed in RIPA buffer (Beyotime) for protein sample preparation or TRIzol (Life Technologies, Carlsbad, CA, USA) for RNA sample preparation. Based on the activation markers for HSCs and vesicle counts per milliliter of culture medium, culture media (CMs) and HSC-derived sEVs were collected after 3 days for the short-term condition, while for the long-term condition, the refreshed culture medium was kept unchanged after 7 days and collected on day 14 to ensure comparable EV numbers per milliliter culture medium.

For rat KC and MO isolation, mononuclear cell-enriched cells were recovered from nonparenchymal liver cells or bone marrow cells flushed from rat femurs by Percoll (GE Healthcare, Princeton, NJ, USA) density gradient centrifugation [21]. The cells obtained were further purified by MACS with anti-CD45-APC and anti-APC microbeads (Miltenyi) according to the manufacturer’s instructions. KCs and MOs were seeded in 25 cm^2^ culture flasks or onto coverslips and cultured in RPMI 1640 medium (Gibco) with 10% FBS (Gibco) at 37 °C in a humidified 5% CO2 atmosphere incubator.

For the isolation of mouse KC, the livers of male C57BL/6J mice were perfused with D-Hanks solution, cut into 2 mm^3^ pieces, and then incubated in digestion solution containing collagenase and pronase E at 37 °C for 30 min to obtain the liver cell suspension. The cell suspension was centrifuged at 50 g for 5 min at 4 °C to remove parenchymal cells, and the nonparenchymal liver cells in the supernatant were collected and subjected to Percoll (GE Healthcare) density gradient centrifugation. KC-enriched cells from the interface of 25% and 60% Percoll were further purified by MACS with anti-CD11b/c-PE and anti-PE microBeads (Miltenyi) according to the manufacturer’s instructions.

### Small extracellular vesicle (sEV) Preparation and identification

The starting volume of the CM for all sEV isolation experiments was 20 mL. CMs were centrifuged at 300 × g for 10 min and then at 21,000 × g for 15 min followed by filtration through 0.22 μm filters (Millipore, Bedford, MA, USA) to remove cell debris and particles larger than 220 nm. The sEVs were pelleted by ultracentrifugation at 100,000 × g for 90 min (CS150GXL, Hitachi, Ltd., Tokyo, Japan). The sEV pellet was washed with 36 mL of PBS, followed by a second ultracentrifugation step at 100,000 × g for 90 min [21]. For an increased recovery rate, sEV fractions were also precipitated by using ExoQuick (System Biosciences, Inc.) according to the manufacturer’s instructions. The pelleted sEV fraction was resuspended in 25 µL of particle-free PBS (Sigma‒Aldrich, St. Louis, MO, USA). All steps were performed at 4 °C. The sEVs from Day 0 to Day 3 HSC CMs were referred to as 3dHSC-sEVs, and those from Day 7 to Day 14 HSC CMs were referred to as 14dHSC-sEVs. In some cases, sEVs were lysed in RIPA buffer (Beyotime) for protein sample preparation or lysed in TRIzol (Life Technologies) for RNA sample preparation. During sEV RNA sample preparation, 50 fmol of Caenorhabditis elegans (cel)-miR-39-3p was spiked in and served as an external control.

The size distribution and concentration of the isolated particles were measured by NTA (NanoSight NS300, Malvern, Worcestershire, UK). The instrument was routinely calibrated by 100 nm polystyrene latex microbeads and particle-free PBS. Samples were diluted 5,000-fold in particle-free PBS. Triplicate measurements were performed for each sample.

The morphology and size of the sEV-enriched particles were examined using transmission electron microscopy (TEM). The sEV fraction was mixed with an equal volume of 4% paraformaldehyde and transferred onto a formvar/carbon-coated nickel grid, followed by incubation for 20 min and three washes in PBS. The grid was fixed with 1% w/v glutaraldehyde for 5 min and washed with distilled water for 2 min. Then, the samples were negatively stained with 4% w/v uranyl acetate for 5 min, embedded in a mixture of 4% uranyl acetate and 2% methyl cellulose (in a volume ratio of 1/9) solution for 10 min on ice in the dark and air-dried. The preparations were examined by TEM (HT7700, Hitachi, Ltd., Tokyo, Japan) at an accelerating voltage of 80.0 kV.

### Proteinase K and RNase protection assays for HSC-sEVs

To investigate the molecules involved in the effect of sEV and distinguish whether they are located outside or inside sEV. Freshly isolated HSC-sEVs were incubated with 40 µg/mL proteinase K (Biosharp, Shanghai, China) at 37 °C for 10 min, followed by addition of 5 mM phenylmethylsulfonyl fluoride (Beyotime) with 10 min incubation at room temperature. Complete proteinase K inactivation was achieved through subsequent heat treatment at 90 °C for 5 min. Subsequently, the sEVs were incubated with 20 µg/mL RNase A (Thermo) at 37 °C for 20 min to eliminate surface-associated RNA. The sEVs were lysed with TRIzol-LS (Life Technologies) before RNA extraction. The control samples were subjected to the same treatments, but PBS was used instead of RNase A [22, 23].

### Coculture of KCs or MOs with HSC-sEVs

The 3dHSC-sEVs and 14dHSC-sEVs were collected as mentioned above and were added to the culture of primary rat KCs or MOs, respectively. The final concentration of sEVs was approximately 1 × 10^11^ particles/mL. For evaluation of the uptake of sEVs by KCs and MOs, HSC-sEVs were labeled with 10 µM SYTO Green Fluorescent Nucleic Acid Stain (Life Technologies) for 20 min at 37 °C and washed three times with 100 kDa MWCO Amicon Ultra Centrifugal Filters (Millipore) to remove the unincorporated dye. The labeled sEVs were suspended in 200 µL of PBS, immediately added to primary KCs or MOs, and incubated for 2–8 h at 37 °C. The flow through from the last wash was added as a control. The cytoskeleton was stained with phalloidin-iFluor 647 (Abcam), and the cellular nuclei were stained with DAPI (Beyotime) for 5 min. Fluorescence was observed using a Zeiss Axio Imager M2 microscope (Carl Zeiss, Oberkochen, Germany). HSC-sEV cocultured KCs or MOs were lysed in TRIzol (Life Technologies) for RNA sample preparation at the indicated time points.

### Immunofluorescence staining and immunohistochemical staining

Immunofluorescence (IF) staining was performed with primary rat HSCs or KCs and MOs grown on coverslips or cryostat sections of mouse liver tissues by incubation with monoclonal antibodies against α-SMA (Abcam, Cambridge, UK), desmin (Abcam), CD68 (Boster Biological Technology, Ltd., Wuhan, China), or CD31 (Abcam) at 37 °C for 1 h. Then, Alexa 488- or Cy3-labeled secondary antibodies (Abcam) were incubated at room temperature for 45 min, and nuclei were counterstained with DAPI (Beyotime). Images were acquired using a Zeiss fluorescence microscope (Zeiss Axio Imager M2, Carl Zeiss, Germany). In some cases, immunohistochemical (IHC) staining was performed on paraffin sections of tissue samples with Envision + kits (Dako, Carpinteria, CA, USA) according to the manufacturer’s instructions. A monoclonal antibody against CD206 (Boster) and polyclonal antibody against Ki67 (Abcam) were used. Images were acquired with an optical microscope (IX51, Olympus, Tokyo, Japan). Detailed information on all antibodies used is provided in the Table S1.

### Western blotting analyses

Protein samples prepared from sEVs, cells or tissues were separated by 10% or 12% sodium dodecyl sulfate‒polyacrylamide gel electrophoresis and transferred onto PVDF membranes (Millipore). The membrane was blocked with 5% nonfat milk for 1 h at room temperature, incubated at 4 °C overnight with primary antibodies, and then incubated with the appropriate HRP secondary antibody for 1 h at room temperature. Total protein staining with Coomassie Brilliant Blue or β-actin served as the loading control. The protein bands were visualized using an enhanced chemiluminescent (ECL) substrate (Tanon, Shanghai, China). Blot images were captured using the ChemiDoc MP Imaging System (Bio-Rad). Detailed information on all antibodies used is provided in Table S1.

### RNA sequencing and data analyses

For mRNA expression profiling, total RNA from 3dHSC- or 14dHSC-sEV-cocultured KCs or MOs was subjected to transcriptome sequencing conducted by OE Biotech Co., Ltd., (Shanghai, China) with three biological repeats for each group. RNA integrity was evaluated using an Agilent 2100 Bioanalyzer (Agilent Technologies, Santa Clara, CA, USA). The libraries were constructed using the TruSeq Stranded mRNA LTSample Prep Kit (Illumina, San Diego, CA, USA) according to the manufacturer’s instructions. Then, these libraries were sequenced on the Illumina sequencing platform (HiSeqTM 2500 or Illumina HiSeq X Ten), and 125 bp/150 bp paired-end reads were generated.

Raw data were processed using Trimmomatic (http://www.usadellab.org/cms/index.php?page=trimmomatic). The reads containing poly-N and the low-quality reads were removed to obtain clean reads. Then, the clean reads were mapped to the reference genome using hisat2 (http://www.ccb.jhu.edu/software/hisat/). The fragments per kilobase of transcript per million mapped reads (FPKM) value of each gene was calculated using cufflinks (http://cufflinks.cbcb.umd.edu/), and the read counts of each gene were obtained by HTSeq-count (http://www-huber.embl.de/HTSeq). Principal component analyses (PCA) were performed with the R statistical Package v.3.0.3 (www.r-project.org). Differentially expressed genes (DEGs) were identified using the DESeq R package (https://bioconductor.org/packages/DESeq/). A p-value < 0.05 and fold change > 2.0 or fold change < 0.5 were set as the threshold for significantly differential expression. Hierarchical cluster analysis of DEGs was performed to explore gene expression patterns. The biological importance of the differentially expressed genes was explored by Ingenuity Pathways Analysis (http://www.ingenuity.com/). Some of the differentially expressed mRNAs were validated by RT-qPCR. Trimmed sequencing reads were deposited in the European Nucleotide Archive (https://www.ebi.ac.uk/biostudies/arrayexpress/studies/E-MTAB-12754).

For miRNA expression profiling, RNA samples from paired 3dHSC-sEVs and 14dHSC-sEVs as well as 3dHSCs and 14HSCs were subjected to small RNA sequencing conducted by OE Biotech Co., Ltd., (Shanghai, China) with three biological repeats for sEV samples and two biological repeats for cell samples. Small RNA libraries were generated using an Illumina TruSeq Small RNA Sample kit (Illumina). Total RNA was subjected to sequential 3ʹ and 5ʹ adapter ligations (T4 RNA Ligase 2, Epicenter) followed by reverse transcription (SuperScript II Reverse Transcriptase, Invitrogen) into a cDNA library. The amplified cDNAs were purified using 6% Novex TBE PAGE gels (Invitrogen), and bands between 147 nt and 157 nt that contained RNA fragments of 22 nt to 30 nt (corresponding to miRNA) were cut out. Library quality was assessed in an Agilent Bioanalyzer 2100 system (Agilent). The qualified cDNA libraries were used for cluster generation and sequenced on the Illumina HiSeq 2500 platform (Illumina) to obtain 125 bp single-end reads.

By using Cutadapt (version 1.7.1) and the FASTX Toolkit (version 0.0.13), adaptors as well as low-quality reads (Q30 < 80%) and low-complexity reads were removed from the raw reads (the total unfiltered reads obtained from the HiSeq 2500 platform), and the remaining reads were designated clean reads. Thereafter, reads shorter than 15 nt or longer than 41 nt were filtered from these clean reads to obtain valid reads. For annotation of the known miRNAs, these filtered reads were subjected to Blast searches against miRNA sequences downloaded from miRBase (Release 22.0). The unmapped reads were aligned against Rfam (version 11.0) using Blastn software to annotate the tRNA, rRNA, snRNA, and Cis-reg species. The reads filtered by Rfam were aligned against the corresponding genome and repeat databases to identify degraded mRNA fragments (gene) and repeats. The expression of miRNAs was normalized as transcripts per million reads (TPM). The miRNAs with a mean expression ≥ 100 (base mean) were regarded as detectable miRNAs. Differentially expressed miRNAs were identified using the DESeq R package (https://bioconductor.org/packages/DESeq/). For inclusion of more varied miRNAs, less stringent criteria were set as the threshold for differential expression (*p* < 0.10 and fold change > 2.0 or fold change < 0.5). Some of the differentially expressed miRNAs were validated by RT-qPCR. Trimmed sequencing reads were deposited in the European Nucleotide Archive (https://www.ebi.ac.uk/biostudies/arrayexpress/studies/E-MTAB-12764).

### Quantitative reverse transcription-polymerase chain reaction (RT-qPCR) analyses

The expression of mRNAs in rat primary KCs and MOs, the human monocyte-like cell line THP-1 and the human stellate cell line LX2 was detected by the PrimeScript™ RT Reagent Kit and TB Green Premix Ex Taq™ Kit (TaKaRa, Dalian, China). Relative gene expression was normalized with β-actin. The RT-qPCR experiments were performed with a StepOnePlus™ Real-Time PCR System (Applied Biosystems, Foster City, CA, USA). The expression of mature miRNAs in HSC whole cell lysates or HSC-sEVs was detected by the miDETECT A Track™ miRNA qRT‒PCR Kit (RiboBio, Guangzhou, China) using the same batch of samples prepared for small RNA sequencing. U6 snRNA served as a reference for cell samples, and spike-in cel-miR-39 served as a reference for sEV samples. The sequences of gene-specific primer pairs and primers for mature miRNAs are provided in Table S2.

### Multicolor flow cytometric analyses

For determination of the phenotypes of primary rat KCs, human THP-1-derived macrophages or primary mouse KCs, multicolor flow cytometric analyses were performed using the following primary antibodies for rats: anti-CD86 (BD Biosciences, San Diego, CA, USA), anti-CD206 (Boster), and anti-CD45 (Miltenyi); anti-CD86 (BioLegend, San Diego, CA, USA), anti-CD206 (BioLegend), and anti-CD45 (BioLegend) for humans; and anti-CD86 (Thermo Fisher Scientific, San Diego, CA, USA), anti-CD206 (Thermo), anti-CD11b (BioLegend), anti-F4/80 (BioLegend), and anti-CD16/32 Fc-receptor blocking antibodies (BioLegend) for mice. Flow cytometry was performed on a BD FACSCalibur system (BD Biosciences, San Jose, CA, USA). Data were analyzed using FlowJo software (TreeStar, Inc., Ashland, OR, USA). Detailed information on all antibodies used is provided in Table S1.

### Transfection of MiRNA mimic or anti-miRNA

Primary rat KCs or human THP-1-derived macrophages were seeded at a density of 2.5 × 10^4^ cells per well in 24-well plates or 2.5 × 10^5^ per 6-cm dish. After 24 h, cells were transfected with 50 nM synthetic miRNA mimic (mimic-99a-5p), miRNA mimic negative control (mimic-NC), anti-miR (anti-99a-5p), or anti-miR negative control (anti-NC) (GenePharma, Shanghai, China) by Lipofectamine 2000 (Invitrogen) according to the manufacturer’s instructions. Eight hours after transfection, some of the cells were collected and subjected to multicolor flow cytometric analyses; some of the cells were lysed in TRIzol (Life Technologies) for RNA sample preparation and subjected to RT-qPCR analyses; and some of the cells were lysed in RIPA buffer (Beyotime) for protein sample preparation and subjected to western blotting analyses.

### Cytokine array analyses

The rat KC or human THP-1 macrophage culture media were collected 24 h after the treatments, then centrifuged at 1,000 × g for 15 min at 4 °C followed by filtration through 0.22 μm filters (Millipore). The relative expression levels of 40 human inflammatory factors in THP-1 macrophage culture media were measured using Human Inflammation Array Q3 (RayBiotech, Norcross, GA, USA) per the manufacturer’s protocol. The relative expression levels of 67 rat inflammatory factors in rat KC culture media were measured using Rat Cytokine Array GS67 (RayBiotech) per the manufacturer’s protocol.

### CCL4-induced mouse chronic liver injury model and primary mice HSCs isolation

For establishment of the chronic liver injury model, male C57BL/6J mice were treated by intraperitoneal injection of CCl4 (0.5 mL/kg, dissolved in olive oil 1:9, v/v) twice a week for 5.5 weeks. Twenty-four hours after each of the last three injections of CCl4, the mice were treated with agomir-99a-5p or antagomir-99a-5p (5 nmol in 150 µL of PBS per mouse, RiboBio), which simulated endogenous miR-99a-5p or served as an inhibitor via the caudal vein. Mice treated with the same dose of corresponding agomir-NC or antagomir-NC (RiboBio) served as controls, and five mice were used in each group. All mice were sacrificed 48 h after the last administration of agomir or antagomir. Blood and livers were collected from each group for further analyses. For observation of the distribution of agomir or antagomir in the liver, the same dose of 5Cy3-labeled agomir-NC or FAM-labeled antagomir-NC was administered via the caudal vein, and the livers were collected 2 h after admission and subjected to immunofluorescence examination as described above.

For liver function tests, blood collected by left ventricular puncture was left undisturbed for 1 h at 37 °C and 2 h at 4 °C. Afterward, the samples were centrifuged at 1000 ×g for 10 min at 4 °C; the clear upper fractions were aliquoted and stored at -80 °C. Serum alanine transaminase (ALT) and aspartate transferase (AST) levels were measured on an ADVIA 1800 autoanalyzer (Siemens Healthcare Diagnostics, Deerfield, IL, USA).

Part of the livers were subjected to primary KC isolation and subsequent flow cytometric analyses for the percentages of CD86- and CD206-positive cells as described above. Part of the livers was preserved in 4% paraformaldehyde, paraffin-embedded and sectioned. The liver tissue sections were stained with hematoxylin and eosin (Beyotime) for routine histology, stained with 0.1% Sirius Red (Sigma‒Aldrich) for collagen evaluation [21], or subjected to IHC staining for the detection of CD206-positive cells. Part of the livers was lysed in RIPA buffer (Beyotime) for protein sample preparation and subjected to western blotting analyses.

Primary murine HSCs were isolated from the livers of male C57BL/6J mice treated with CCL4 for 4 weeks or control. HSC-enriched cells were obtained from nonparenchymal liver cells by density gradient centrifugation as described previously [19]. As shown by the intracellular autofluorescence of vitamin A and trypan blue exclusive assay, the purity and vitality of the purified HSCs were both above 98%. HSCs were seeded in 25 cm^2^ culture flasks or onto coverslips and cultured in DMEM (Gibco) with 10% Exo-free FBS (System Biosciences, Inc.) at 37 °C in a 5% CO2 atmosphere incubator. HSCs were collected and lysed in TRIzol (Life Technologies) for RNA sample preparation. The culture media (CMs) were collected at 24 h for isolation of HSC-derived sEVs.

### Colony formation assays

Human THP-1-derived macrophages were pretreated with mimic-99a-5p or mimic-NC (GenePharma) for 8 h as described above. HepG2 or Huh7 cells were seeded at 300 cells per well in 12-well plates, and 60 pretreated THP-1 cells were added to each well. The cells were maintained in DMEM containing 10% FBS (Gibco) at 37 °C for 7 days. Additional culture medium was added every 3 days. On Day 8, the cells were fixed with 2% formalin and stained with 0.5% crystal violet (Sigma‒Aldrich) solution for 10 min. Stained cells were rinsed with ddH2O. Cell colonies with more than 50 cells were counted under a microscope (Olympus).

### Cell-line-derived xenograft mouse model

For establishment of the nude mouse xenograft model of HCC, twenty 5-week-old male BALB/c nude mice were divided into four groups, and each group contained 5 mice. HepG2 cells were inoculated hypodermically into the forelimbs of mice with or without THP-1 cells. Each mouse was injected with 1 × 10^6^ HepG2 cells alone, 1 × 10^6^ HepG2 cells mixed with 2 × 10^5^ untreated THP-1 cells, 2 × 10^5^ mimic-99a-5p (GenePharma)-pretreated THP-1 cells, 2 × 10^5^ mimic-NC (GenePharma)-pretreated THP-1 cells, 2 × 10^5^ anti-99a-5p (GenePharma)-pretreated THP-1 cells, or 2 × 10^5^ anti-NC (GenePharma)-pretreated THP-1 cells. The size of the tumor was measured every other day. All mice were euthanized at Day 28, and tumors were excised and weighed. Part of the tumors was preserved in 4% paraformaldehyde, paraffin-embedded and sectioned. The tumor tissue sections were subjected to IHC staining for the detection of Ki67-positive cells. Part of the tumors was lysed in RIPA buffer (Beyotime) for protein sample preparation and subjected to western blotting analyses.

### Luciferase assays

The putative miR-99a-5p anchor element from 2229 to 2266 of CD93 mRNA (NM_012072.4, 5’- AAAGGCCCCTTGGAACATGCAGGTATTTTCTA**CGGG**TG-3’) was termed the miR-99a-5p recognition element (CD93MRE99). The synthesized CD93 3’UTR fragment containing three copies of CD93MRE99 with flanking XhoI and NotI restriction enzyme cohesive ends was annealed and ligated into the psiCHECK-2 vector (Promega, Madison, WI, USA) between the XhoI and NotI sites located 3’ to the Renilla luciferase translational stop codon, resulting in a single insertion as psiCHECK-2/CD93MRE99 × 3 [24]. A mutant vector in which the nucleotide bases “CGGG” within CD93MRE99 were replaced by “GCCC” was also constructed and termed psiCHECK-2/CD93MRE99mut×3. The post-transcriptional regulation of Renilla luciferase was potentially mediated by CD93MRE99. The activity of Renilla luciferase was normalized to the internal firefly luciferase activity. The nucleotide sequences of the constructed plasmids were confirmed by DNA sequencing (Sangon Biotech, Shanghai, China).

HEK 293T cells were seeded in 24-well plates 24 h before transfection and transiently cotransfected with psiCHECK-2/CD93MRE99 × 3 or psiCHECK-2/CD93MRE99 × 3 and mimic-99a-5p or mimic-NC (GenePharma) using Lipofectamine 2000 (Invitrogen). Luciferase assays were performed 48 h later using a dual-luciferase reporter assay kit (Promega). Renilla and firefly luciferase signals were detected using a Veritas Microplate Luminometer (Turner Biosystems, Sunnyvale, CA, USA).

### Transfection of CD93 SiRNA and CD93 overexpression plasmid

Three siRNA fragments targeting CD93 and negative control siRNA (Genepharma) were transfected into THP-1-derived macrophages by Lipofectamine 2000 Reagent (Invitrogen) at a final concentration of 50 nM. THP-1 cells were collected 24 h after transfection, and lysed in RIPA buffer (Beyotime) for protein sample preparation or TRIzol (Life Technologies) for RNA sample preparation. The knocking down efficiency of siRNA was determined by RT-qPCR. The CD93 siRNA of the highest knocking down efficiency was used in subsequent experiments.

The CD93 overexpression plasmids (pLV3-CMV-CD93(human)-CopGFP-Puro and pLV3-CMV-Cd93(rat)-CopGFP-Puro, OE-CD93) or negative control (pLV3-CMV-MCS-3×FLAG-CopGFP-Puro, OE-NC) were obtained from MiaoLingBio (MiaoLingBio, Wuhan, China). Plasmids were transfected into THP-1-derived macrophages or primary rat KCs cultured in 6-well plates by Lipofectamine 2000 Reagent (Invitrogen) at a final concentration of 2.5 µg per well. Cells and culture media were collected 24 h after transfection. Cells were lysed in RIPA buffer (Beyotime) for protein sample preparation or TRIzol (Life Technologies) for RNA sample preparation. Culture media were saved for cytokine array analyses.

### Statistical analyses

Unless otherwise specified, statistical analyses were performed with GraphPad Prism 7.0 software (GraphPad Software, Inc., La Jolla, CA). Quantitative data are expressed as the means ± standard errors of the means (SEMs). Comparisons between groups were made by Student’s t test or one-way ANOVA. Correlations between two variables were determined using Pearson’s correlation analysis. Statistical significance was defined as *p* < 0.05. Except for small RNA sequencing of primary HSCs, which included two biological replicates for each group, the data provided in the present study came from three or more independent experiments.

The miRNA sequencing data and survival information of 362 HCC patients were obtained from The Cancer Genome Atlas (TCGA) dataset (https://portal.gdc.cancer.gov/). Kaplan–Meier survival curves with log-rank tests were plotted with GraphPad Prism 7.0 (GraphPad Software, La Jolla, CA, USA) to compare the survival difference between patients with higher and lower individual miRNA expression, and a 33rd percentile cutoff was adopted. A log-rank *p*-value < 0.05 was considered statistically significant.

The RNA sequencing data for of 371 HCC patients were obtained from TCGA dataset. The correlation between the mRNA expression of CD93 and CD206 in human HCC tissues was determined by Spearman’s correlation analysis. A *p*-value < 0.05 was considered statistically significant.

## Results

### Isolation and characterization of short-term and long-term activated HSCs and their sEVs

**Fig. 1:**
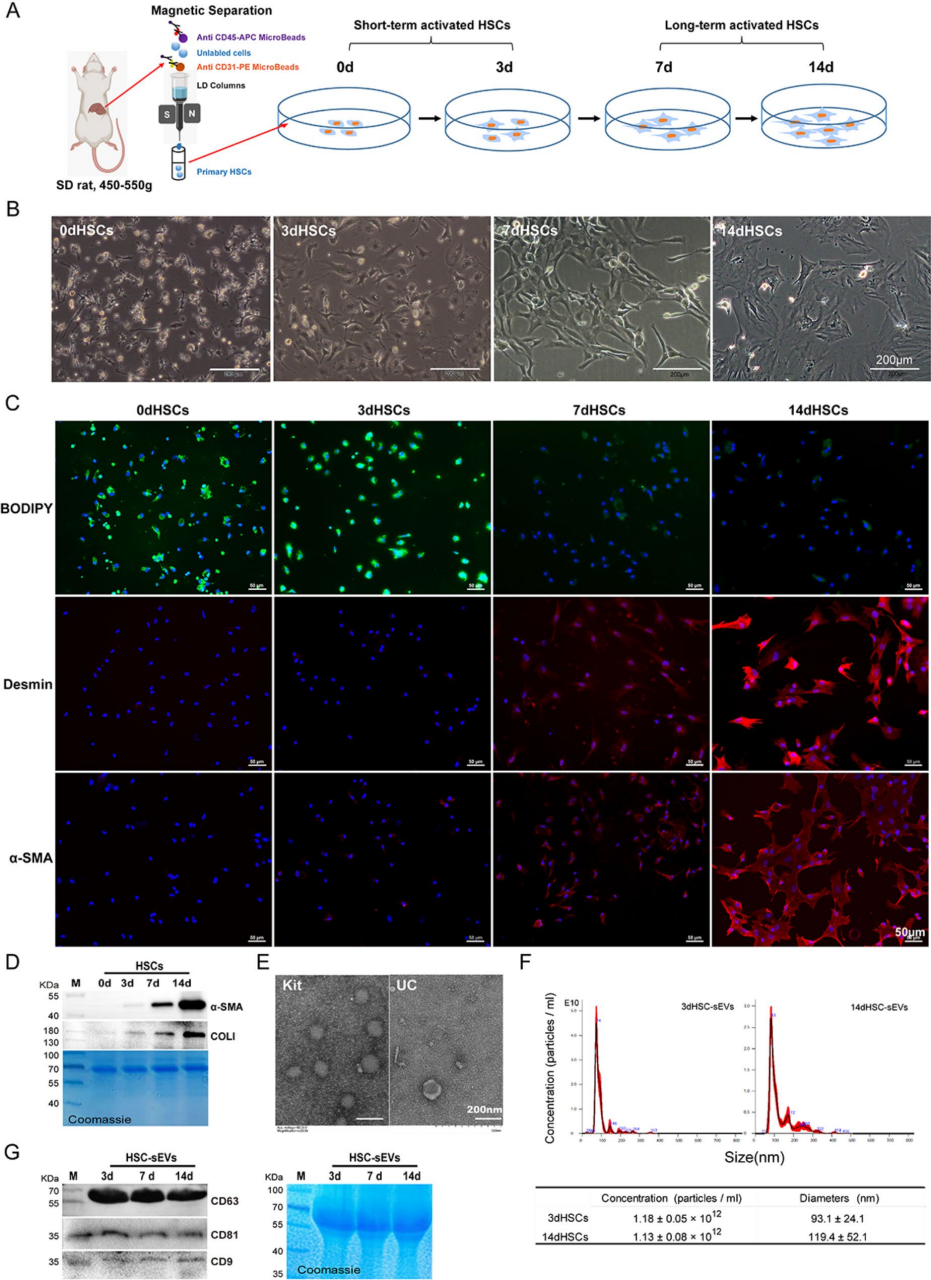
Characterization of culture-activated primary rat HSCs and sEVs isolated from the culture media. (**A**) Schematic representation of the process for rat primary hepatic stellate cell isolation and culture. (**B**) Morphology of primary hepatic stellate cells of rats at Day 0, Day 3, Day 7 and Day 14, bright field, scale bar = 200 μm. (**C**) *BODIPY 493/503* staining of cytoplasmic lipid droplets and immunofluorescence staining of Desmin and α-smooth muscle actin (α-SMA) of HSCs at the indicated time points. *BODIPY 493/503 (green)*,* Desmin and* α-SMA *(Cy3*,* red)*, nuclei were counterstained with DAPI (blue), scale bar = 50 μm. (**D**) Western blotting analyses of the HSC activation markers, α-SMA and collagen type I (ColI). Cells were collected at the indicated time points. Coomassie blue staining was used as a loading control. (**E**) Transmission electron microscopy image of the particles isolated from the primary HSC culture medium. Left, sEVs enriched by the ExoQuick Kit; right, sEVs enriched by ultracentrifugation (UC), scale bar = 200 nm. (**F**) Representative size distribution of isolated particles and their concentrations determined by nanoparticle tracking analyses. (**G**) The expression of CD63, CD81 and CD9 in the isolated particles detected by western blotting. *Coomassie blue staining was used as a loading control.* sEV, small extracellular vesicle; 3dHSCs, primary hepatic stellate cells cultured on Day 3; and 14dHSCs, primary hepatic stellate cells cultured on Day 14

To comprehensively investigate how the activation state of HSCs affects the biological composition and functional properties of sEVs, we designed an in vitro time-course experiment using purified primary rat HSCs (Fig. 1A). These cells were seeded and continuously cultured on plastic surface for up to 14 days to monitor their progressive activation (Fig. 1B, C and *D*, Figure S1).

Based on characteristic morphological changes (Fig. 1B), cytoplasmic lipid droplets and molecular marker expression patterns (Fig. 1C and *D*), we categorized HSCs into two activation states*.* Freshly isolated HSCs were defined as quiescent HSCs (0dHSCs), identified by their abundant cytoplasmic lipid droplets. HSCs cultured for up to three days were defined as short-term activated HSCs (3dHSCs), remained rich in lipid droplet content and slightly increased expression of activation markers, including α-smooth muscle actin (α-SMA) and collagen type I (COLI)*.* HSCs cultured for seven days or longer (up to 14 days) were classified as long-term activated HSCs (14dHSCs), with diminished lipid droplets and significantly increased α-SMA, COLI and intermediate filament Desmin, representing a fully activated phenotype*.*

Culture media (CMs) from Day 0 to Day 3 culture and from Day 7 to Day 14 culture were collected as short-term activated HSC media (3dHSC-CMs) and long-term activated HSC media (14dHSC-CMs), respectively. The sEVs were then isolated from these culture media, designated as short-term activated HSC sEVs (3dHSC-sEVs) and long-term activated HSC sEVs (14dHSC-sEVs). Characterization of these isolated particles sEVs by electron microscopy revealed their spherical morphology (Fig. 1E), and size distribution primarily smaller than 200 nm in diameter. Additionally, quantification of particles numbers showed comparable yields from both 3dHSC-CMs and 14dHSC-CMs per milliliter of media (Fig. 1F). To confirm the identity of the isolated sEVs, western blot analysis demonstrated the presence of the canonical exosomal markers CD63, CD81, and CD9 (Fig. 1G).

In conclusion, our study refined the classification of HSC activation states, establishing clear distinctions between short-term and long-term activated HSCs. By successfully isolating and characterizing their corresponding sEVs, we laid the groundwork for investigating their distinct roles in modulating liver macrophage polarization.

### HSC-derived sEVs differentially regulate the polarization of liver macrophage subgroups depending on the activation stage of the HSCs

To systematically examine whether sEVs from different activation stages of HSCs exert distinct effects on liver macrophage polarization, we designed experiments targeting the two primary macrophage subpopulations in the liver: resident macrophages (Kupffer cells, KCs) and infiltrated bone marrow-derived monocytes/macrophages (MOs) [25]. Given the established differences in macrophage polarization [26, 27], with KCs being more prone to M2-like differentiation and MOs exhibiting a more proinflammatory M1-like phenotype, we sought to determine how sEVs from short-term versus long-term activated HSCs influence these macrophage subsets.

Canonical pathway analyses of the differentially expressed genes between untreated KCs and MOs revealed an activated Th2 and suppressed PD-1/PD-L1 pathway in KCs compared to MOs (Table S3)51, supporting previous findings that KCs naturally lean toward an M2 phenotype, while MOs are more inclined toward M1 polarization [26, 27].

**Fig. 2:**
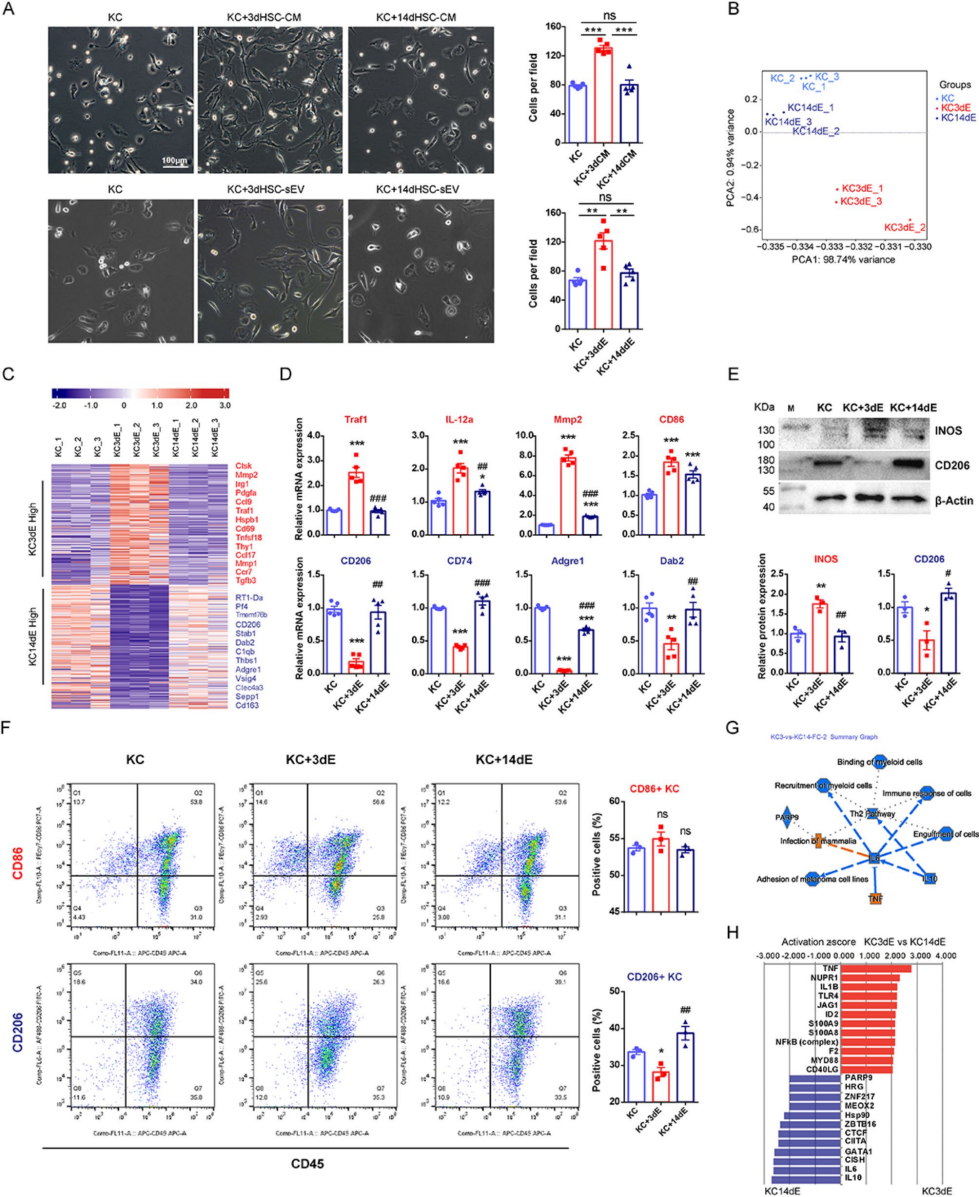
The effects of short-term activated HSC (3dHSC)- and long-term activated HSC (14dHSC)-derived conditioned medium (CM) and sEVs on liver macrophage (KC). (**A**) Morphology of HSC-sEV-cocultured KCs. Gene expression profiles of 3dHSC- and 14dHSC-sEV-treated KCs were obtained by RNA-Seq. (**B**) Principal component analyses (PCA) based on the gene expression profiles of 3dHSC- and 14dHSC**-**derived sEV-treated liver resident macrophages as well as untreated KCs obtained by RNA-Seq. Each dot represents one RNA-Seq dataset of primary cells from one rat. (**C**) A heatmap was generated on the set of 388 differentially expressed genes between the 3dHSC- and 14dHSC-sEV-treated KCs. Representative genes are listed on the right side (P value < 0.05 and fold change > 2.0 or fold change < 0.5). The scale represents normalized log2 gene expression levels. (**D**) The gene expression levels of key macrophage biomarkers in KCs cocultured with 3dHSC- or 14dHSC-sEVs were determined by RT-qPCR using the same batch of samples used in RNA-Seq. (**E**) The protein expression of INOS and CD206 in KCs cocultured with 3dHSC- or 14dHSC-sEVs was detected by western blotting. (**F**) Surface marker expression in KCs cocultured with 3dHSC- or 14dHSC-sEVs was determined by flow cytometry. (**G**) The major biological activities for the 388 differentially expressed genes between 3dHSC- and 14dHSC-sEV-cocultured KCs are provided as a summary graph by Ingenuity Pathways Analysis. Orange, activated in KC3dE; blue, inhibited in KC3dE. (**H**) Upstream regulators of the 388 differentially expressed genes between 3dHSC- and 14dHSC-sEV-cocultured KCs. Red, activated KCs cocultured with 3dHSC-sEVs; green, activated KCs cocultured with 14dHSC-sEVs (*p* < 0.05 and activation z score > 2 or < -2). Statistical significance was determined by Student’s t test relative to untreated KCs, *** *p* < 0.001, ** *p* < 0.01, * *p* < 0.05; and Student’s t test relative to 3dHSC-sEV-cocultured KCs, ### *p* < 0.001, ## *p* < 0.01, # *p* < 0.05

To directly assess the effects of HSC-secreted factors, primary rat KCs (Figure S2) were cocultured with either 3dHSC- or 14dHSC-CM for 48 h. Compared to 14dHSC-CM, 3dHSC-CM increased the number of adherent KCs and induced a more spindle-shaped, outspread morphology, whereas KCs treated with 14dHSC-CM remained a polygonal and spheroid-like appearance (Fig. 2A).

To determine whether these effects were mediated by sEVs, we isolated sEVs from 3dHSC- and 14dHSC-CM and cocultured them with KCs. KCs can effectively uptake HSC-sEVs (Figure S3). Notably, the morphological changes induced by 3dHSC- and 14dHSC-sEVs closely resembled those observed in CM-treated KCs, confirming that sEVs are responsible for these effects (Fig. 2A).

To further investigate how 3dHSC- and 14dHSC-sEVs differentially regulate KC polarization, we performed RNA sequencing on KCs after coculture with these sEVs. Principal component analyses (PCA) showed that untreated KCs clustered closely with 14dHSC-sEV-treated KCs, while 3dHSC-sEV-treated KCs formed a distinct cluster, suggesting a significant transcriptomic shift (Fig. 2B).

A total of 388 genes were differentially expressed (fold change > 2.0 or fold change < 0.5, *p* < 0.05) between KCs treated with 3dHSC- versus 14dHSC-sEVs, as listed in Table S4. Heatmap based on these genes further confirmed the similarity between untreated and 14dHSC-sEV-treated KCs, while 3dHSC-sEV-treated KCs exhibited a unique gene expression profile, consistent with PCA results (Fig. 2C).

Strikingly, 3dHSC-sEVs treatment increased the expression of M1-associated proinflammatory genes, including Mgll, Irg1, Traf1, Ccl9, and Ccr7 [28, 29, 30, 31, 32], as well as genes involved in extracellular matrix degradation, such as Ctsk, Mmp2 and Mmp10 [33, 34] (Fig. 2C, Table S4). Conversely, 3dHSC-sEV treatment downregulated hallmark M2 macrophage markers, including Mrc1 (CD206), CD163, C1qb, Adgre1 (F4/80), CD74, Clec10a, Dab2, and Stab1 [28, 35, 36, 37, 38] (Fig. 2C, Table S4). To validate these findings, we conducted RT-qPCR (Fig. 2D), western blotting (Fig. 2E), and flow cytometry (Fig. 2F), all of which confirmed the differential expression of M1 and M2 macrophage markers. To assess the biological importance of the differentially expressed genes, Ingenuity Pathways Analysis *(IPA*, www.ingenuity.com*) *was performed. Activated pathways in the 3dHSC-sEV-treated KCs were strongly associated with TNF signaling and infection response, whereas inhibited pathways were linked to TH2 differentiation, which promotes M2 macrophage polarization* (*Fig. 2G*)*. Upstream regulator analysis further suggested that 3dHSC-sEV treatment activated M1- associated factors (TNF, IL1B, JAG1, etc.), while 14dHSC-sEV treatment activated M2- associated factors (IL-10, IL-6, CIITA, GATA1, etc.) (Fig. 2H).

**Fig. 3:**
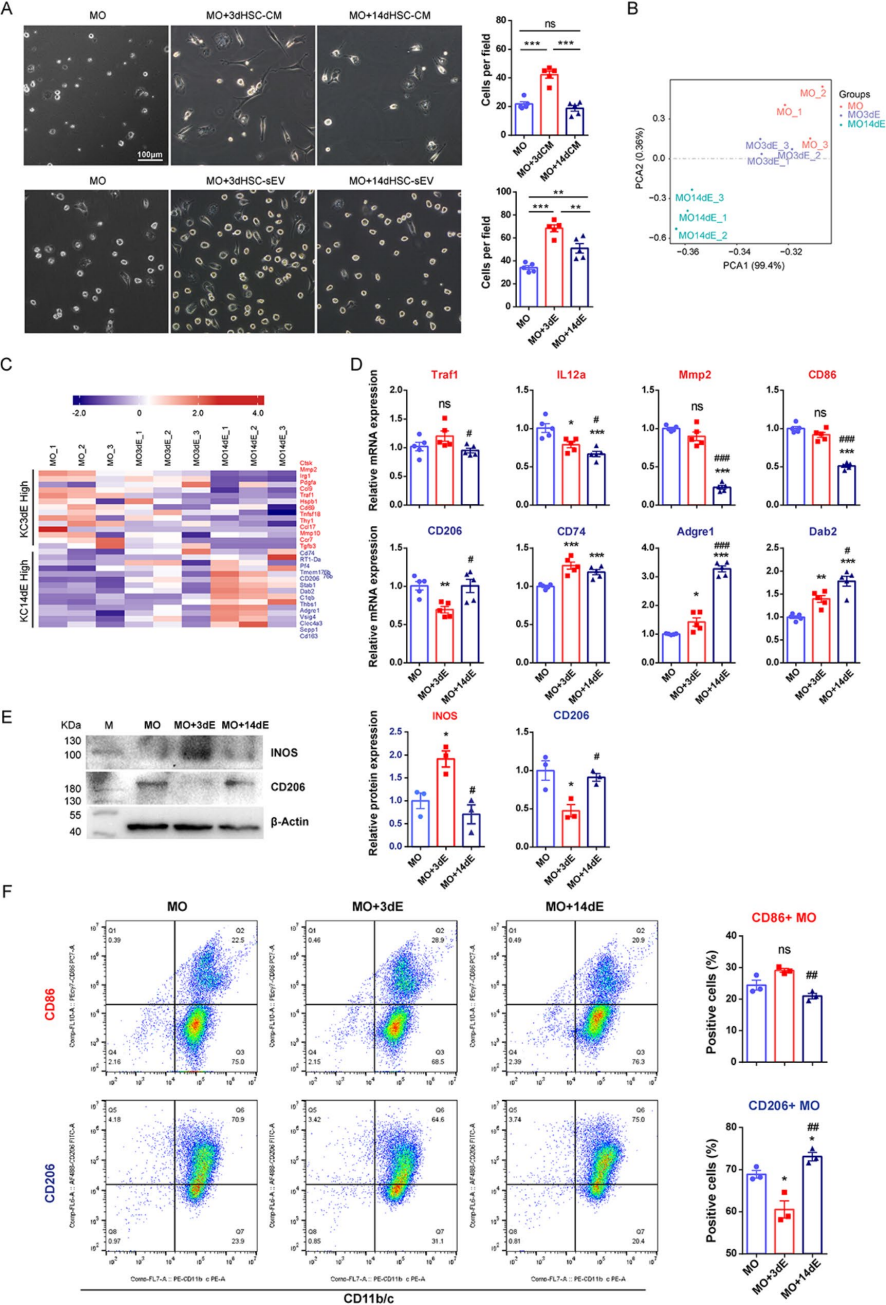
The effects of short-term activated HSC (3dHSC)- and long-term activated HSC (14dHSC)-derived conditioned medium (CM) and sEVs on bone marrow-derived monocyte (MO) differentiation. Primary rat MOs were cocultured with 3dHSC- and 14dHSC-CM or sEVs; untreated cells served as controls. (**A**) Morphology of HSC-sEV-cocultured MOs, scale bar = 100 μm. (**B**) Principal component analysis (PCA) based on the gene expression profiles of 3dHSC- and 14dHSC-sEV-treated MOs as well as untreated MOs obtained by RNA-Seq. Each dot represents one RNA-Seq dataset of primary cells from one rat. (**C**) Heatmaps were generated on a set of 28 representative genes that were differentially expressed between 3dHSC- and 14dHSC-derived sEV-treated Kupffer cells (KCs), as listed in Fig. 2C (P value < 0.05 and fold change > 2.0 or fold change < 0.5); red, highly expressed in 3dHSC-sEV-cocultured KCs; green, highly expressed in 14dHSC-sEV-cocultured KCs. Each row represents an individual gene, and each column represents an individual sample. The scale represents normalized log2 gene expression levels. (**D**) The gene expression levels of key macrophage biomarkers in MOs cocultured with 3dHSC- or 14dHSC-sEVs were determined by RT‒qPCR using the same batch of samples in RNA-Seq. The relative gene expression levels were normalized to β-Actin and untreated MOs. (**E**) The protein expression of INOS and CD206 in MOs cocultured with 3dHSC- or 14dHSC-sEVs was detected by western blotting. (**F**) Surface marker expression in MOs cocultured with 3dHSC- or 14dHSC-sEVs was determined by flow cytometry. Statistical significance was determined by Student’s t test relative to untreated MOs, *** *p* < 0.001, ** *p* < 0.01, * *p* < 0.05; Student’s t test relative to 3dHSC-sEV-cocultured MOs, ### *p* < 0.001, ## *p* < 0.01, # *p* < 0.05, ns, not significant

To extend these findings, we evaluated the effects of 3dHSC- and 14dHSC-sEVs on MOs, using the same experimental approach as for KCs (Fig. 3, Figure S2, S3). Similar to KCs, MOs treated with 3dHSC-CMs or sEVs exhibited increased adhesion and an elongated morphology, whereas 14dHSC-sEV-treated MOs retained a rounded shape (Fig. 3A). PCA analysis showed that the MOs cocultured with 3dHSC-sEVs clustered closer to untreated MOs, while 14dHSC-sEV-treated MOs formed a distinct cluster (Fig. 3B). Furthermore, 14dHSC-sEV-treated MOs exhibited increased expression of key M2 markers, including CD206, CD163, C1qb, Adgre1, CD74, Dab2 and Stab1 (Fig. 3C), suggesting that long-term activated HSC-derived sEVs promote an M2-like macrophage phenotype*.* The differential expression of macrophage biomarkers in each group was further validated by RT-qPCR (Fig. 3D), western blotting (Fig. 3E), and flow cytometric assays (Fig. 3F).

Our findings reveal a distinct regulatory role of HSC-derived sEVs in macrophage polarization, which is dependent on the activation stage of the HSCs*.* Short-term activated HSC-derived sEVs (3dHSC-sEVs) drive KCs toward an M1-like proinflammatory state by increasing M1 marker expression and inhibiting M2-associated genes. While long-term activated HSC-derived sEVs (14dHSC-sEVs) lack this proinflammatory capacity and instead promote M2-like polarization in MOs. This suggests that the activation stage of HSCs dynamically influences the immune environment via sEV-mediated macrophage reprogramming, potentially impacting liver inflammation and fibrosis progression*.*

### Differential MiRNAs transported by the short-term and long-term activated HSC- sEVs

To investigate the molecular mechanisms underlying the distinct effects of short-term activated and long-term activated HSC-sEVs on macrophages polarization. We carried out proteinase K protection assays for HSC-sEVs.Proteinase K treated HSC-sEVs showed similar effects on KCs as untreated HSC-sEVs (Figure S4) which emphasized the biological significance of the content protected by sEVs*.* We then focused on miRNAs, the key functional cargoes of sEVs that regulate gene expression in recipient cells [39]. We hypothesized that differences in miRNA content between 3dHSC-sEVs and 14dHSC-sEVs may explain their differential effects on immune modulation*.*

**Fig. 4:**
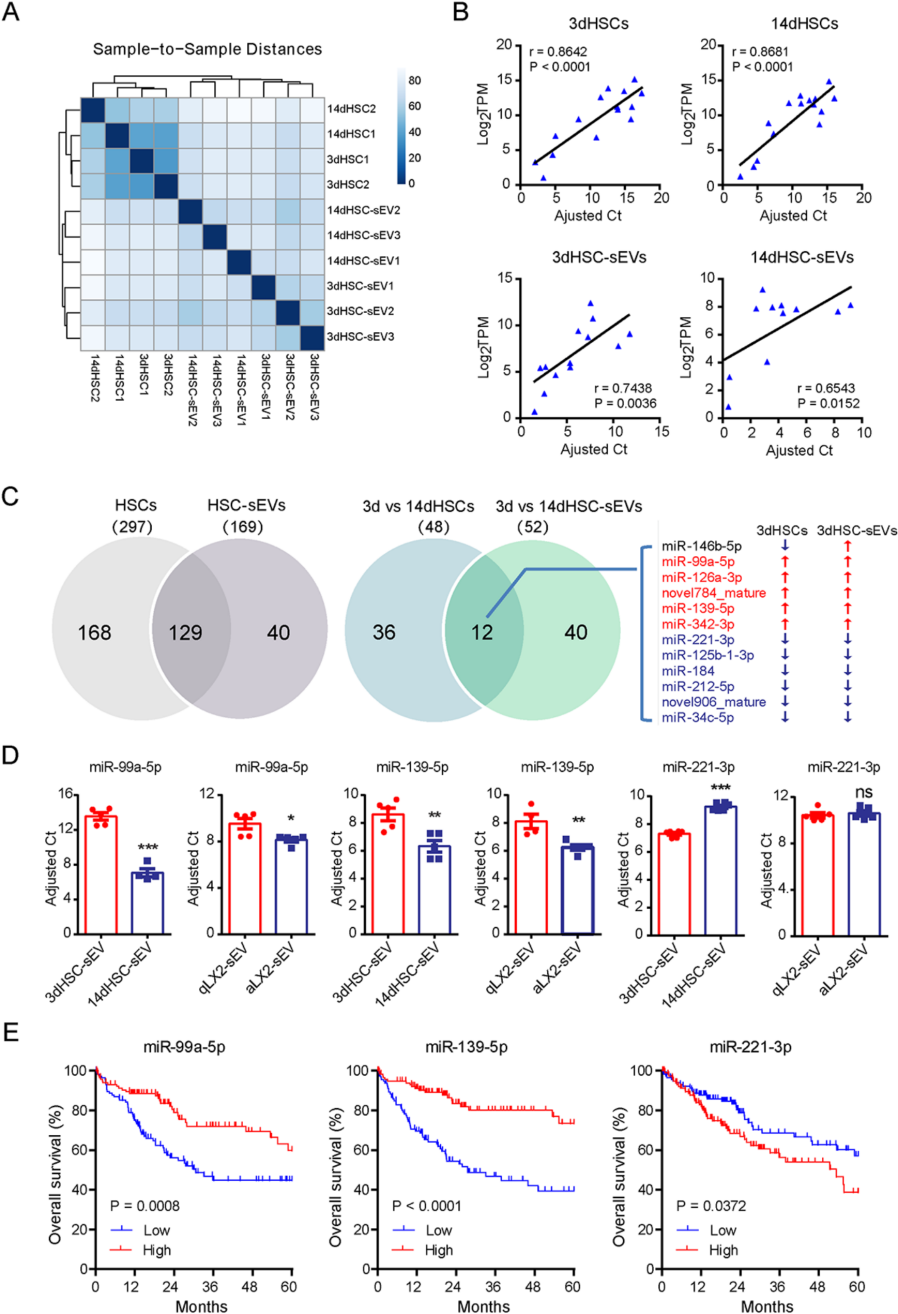
The miRNA profiling and validation of short-term activated HSCs (3dHSCs) and long-term activated HSCs (14dHSCs) and corresponding sEVs. (**A**) Heatmap generated with DeSeq2 software packages showing the Euclidean distances between the samples. (**B**) Correlation of normalized reads (log_2_TPM) obtained by small RNA-Seq and adjusted RT-qPCR CT values for 15 miRNAs (rno-let-7f-5p, rno-miR-10a-5p, rno-miR-125a-5p, rno-miR-125b-1-3p, rno-miR-127-3p, rno-miR-139-5p, rno-miR-143-3p, rno-miR-146b-5p, rno-miR-27a-3p, rno-miR-27b-3p, rno-miR-30a-5p, rno-miR-30b-5p, rno-miR-423-5p, rno-miR-486, and rno-miR-99a-5p). RT-qPCR was performed with the same batch of RNAs prepared for sequencing. The relative expression levels of the selected miRNAs as determined by qPCR were expressed as adjusted Ct (ΔCt + 15) for cell samples and as (ΔCt + 25) for sEV samples. U6snRNA served as the reference gene for cell samples, and spike-in cel-miR-39 served as the reference gene for sEV samples. (**C**) Venn diagram showing the common and unique miRNAs identified in HSCs and HSC sEVs; the differentially expressed miRNAs between 3dHSCs and 14dHSCs and those between the corresponding sEVs were compared; only those miRNAs with a base mean > 100 were included (Table S6). The 12 miRNAs expressed differentially in either HSCs or the corresponding sEVs upon activation are listed on the right side; the up- or downregulation of these miRNAs is indicated by an upward red arrow or downward green arrow. (**D**) The expression of miR-99a-5p, miR-139-5p, and miR-221-3p in sEVs from culture-activated rat primary HSCs and the TGF-β-activated human hepatic stellate cell line LX2 was determined by RT-qPCR, and spike-in cel-miR-39 served as a reference. The relative expression of miRNA was expressed as adjusted Ct (40 - Ct), and statistical significance was determined by Student’s t test, *** *p* < 0.001, ** *p* < 0.01, * *p* < 0.05, ns, not significant. (**E**) Overall survival analyses of miR-99a-5p, miR-139-5p, and miR-221-3p expression in HCC patients based on TCGA survival data, and a 33rd percentile cutoff was adopted

To test this hypothesis, we performed small RNA sequencing on HSCs and their corresponding sEVs at different activation stages, including 3dHSCs, 14dHSCs, 3dHSC-sEVs, and 14dHSC-sEVs (Table S5). Biological reproducibility was confirmed via correlation analysis of normalized miRNA reads, demonstrating a high degree of consistency across replicates (Fig. 4A). To validate the sequencing results, we performed RT-qPCR analyses on a panel of 15 differentially expressed miRNAs (let-7f-5p, miR-10a-5p, miR-125a-5p, miR-125b-1-3p, miR-127-3p, miR-139-5p, miR-143-3p, miR-146b-5p, miR-27a-3p, miR-27b-3p, miR-30a-5p, miR-30b-5p, miR-423-5p, miR-486, and miR-99a-5p). The relative expression levels measured by RT-qPCR correlated well with the normalized sequencing reads, confirming the reliability of our data (Fig. 4B).

Through this analysis, we identified 129 miRNAs common to both HSCs and their corresponding sEVs, while 168 miRNAs were exclusively present in HSCs, and 40 were unique to HSC-sEVs (Fig. 4C, Table S6). To further refine our analysis, we further identified differentially expressed miRNAs in both HSCs and their secreted sEVs during activation, applying criteria of base mean > 100, fold change > 2.0 or < 0.5 and *p* < 0.10. We found that 48 miRNAs were differentially expressed in 3dHSCs compared to 14dHSCs (Table S7), and 52 miRNAs were differentially expressed in 3dHSC-sEVs compared to 14dHSC-sEVs (Table S8).

Notably, only 12 miRNAs showed differential expression in both HSCs and their corresponding sEVs during activation, suggesting that most miRNAs are selectively packaged into sEVs rather than passively reflecting intracellular miRNA levels. Of these 12 miRNAs, 11 exhibited consistent expression trends between cells and sEVs, except for miR-146b-5p, which showed a discordant pattern.

Based on their expression patterns, we classified these miRNAs into two distinct groups: “short-term activated HSC-sEV-specific miRNAs”: These five miRNAs (miR-99a-5p, miR-126a-3p, novel784_mature, miR-139-5p, miR-342-3p) were enriched in 3dHSCs and 3dHSC-sEVs but downregulated in 14dHSCs and 14dHSC-sEVs. “Long-term activated HSC-sEV-specific miRNAs”: These six miRNAs (miR-221-3p, miR-34c-5p, miR-125b-1-3p, miR-184, miR-212-5p, novel906_mature) exhibited the opposite trend, being upregulated in 14dHSCs and 14dHSC-sEVs but downregulated in 3dHSCs and 3dHSC-sEVs (Fig. 4C).

**Fig. 5:**
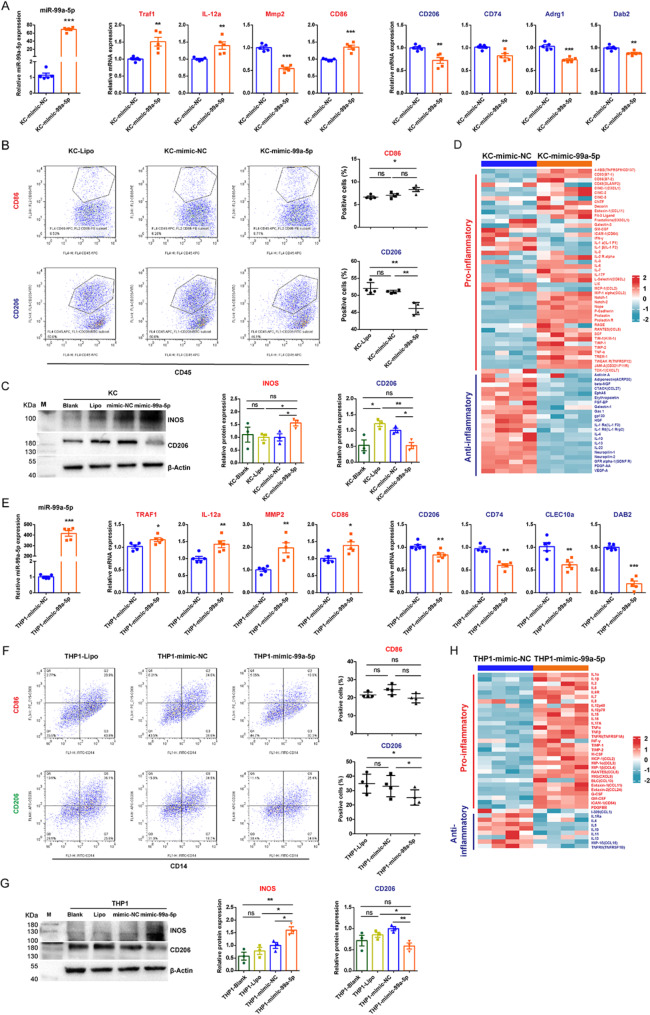
The effects of short-term activated HSC-sEV-specific miR-99a-5p on primary rat liver macrophage (KC) and human THP-1 macrophage differentiation. (**A**, **E**) The expression of miR-99a-5p and the mRNAs of 8 genes associated with HSC-sEV-induced macrophage differentiation in miR-99a-5p mimic-treated primary rat KCs (**A**) and human THP-1 macrophages (**E**) was determined by RT-qPCR. The relative miR-99a-5p expression was normalized to that of U6snRNA, and the relative gene expression was normalized to that of 18 S rRNA (or β-Actin) and then to miRNA mimic negative control (mimic-NC). Red font, highly expressed in 3dHSC-sEV cocultured KCs, green font, highly expressed in 14dHSC-sEV cocultured KCs. (**B**, **F**) Surface marker expression in miR-99a-5p mimic-treated primary rat KCs (**B**) and human THP-1 macrophages (**F**) was determined by flow cytometry. Representative flow cytometry images and statistical histograms for CD86- and CD206-positive cells in each group are provided. (**C**, **G**) The protein expression of INOS and CD206 in miR-99a-5p mimic-treated primary rat KCs (**C**) and human THP-1 macrophages (**G**) was detected by western blotting, β-Actin served as a loading control and was normalized to mimic-NC treated cells. (**D**, **H**) Cytokine array analyses for culture media from primary KCs (**D**) and human THP-1 macrophages (**H**) treated by miR-99a-5p mimic and control (mimic-NC). Inflammatory factors are listed on the right side. Each row represents an individual inflammatory factor, and each column represents an individual sample. Red font, proinflammatory factors; green font, anti- inflammatory factors. The scale represents normalized log2 inflammatory factor expression levels. Statistical significance was determined by Student’s t test, *** *p* < 0.001, ** *p* < 0.01, * *p* < 0.05

To confirm these findings, we performed RT-qPCR validation using sEVs from both culture-activated primary rat HSCs and the TGF-β1-activated human HSC cell line LX2 (Figure S6A and B). The expression data were consistently reproduced the differential miRNA expression trends observed in our sequencing data (Fig. 4D. RNase protection assays further confirmed that these miRNAs were protected by the sEV lipid bilayer* (*FigureS5).

To evaluate the pathological relevance of these differentially expressed miRNAs, we performed survival analyses using miRNA expression and patient survival data from The Cancer Genome Atlas (TCGA, https://portal.gdc.cancer.gov/). Our analysis revealed a significant correlation between specific HSC-sEV miRNAs and patient prognosis in HCC. Lower expression of two short-term activated HSC-sEV-specific miRNAs (miR-99a-5p and miR-139-5p) and higher expression of one long-term activated HSC-sEV-specific miRNA (miR-221-3p) were associated with poorer patient prognosis (Fig. 4E).

These findings suggest that short-term activated HSC-sEVs contain unique miRNAs that may contribute to immune activation and be linked to better patient prognosis, whereas long-term activated HSC-sEVs may promote an immunosuppressive microenvironment associated with poorer outcomes.

### The short-term activated HSC-sEV-specific miR-99a-5p induces M1-like macrophages and reduces liver fibrosis

We investigated the biological relevance of the short-term activated HSC-sEV-specific miRNAs in macrophage differentiation, focusing on miR-99a-5p, the most abundant and highly expressed miRNA in short-term activated rat 3dHSC-sEVs and untreated human LX2-sEVs. It accounts for 4.03% of the total reads of all the 3dHSC-sEV-miRNAs detected, and is the fifth most abundant miRNA in 3dHSC-sEV (Table S9, Fig. 4D). Additionally, miR-99a-5p was found to be highly expressed in HSCs compared to human macrophages, liver cells, and HCC cells (Figure S6C).

To explore its role in macrophage differentiation, primary rat KCs and PMA-differentiated human THP-1 macrophages were treated with lipid nanoparticles loaded with miR-99a-5p mimic to directly deliver and overexpress miR-99a-5p. We assessed the expression of genes associated with HSC-sEV-induced macrophage differentiation (Table S4) using RT-qPCR, flow cytometry, and western blotting. The results revealed that M2-related genes were downregulated in both cell types treated with the miR-99a-5p mimic, including CD206 (Mrc1), CD74, Adgre1 (F4/80), and Dab2 in primary rat KCs (Fig. 5A, B, C), and CD206, CD74, CLEC10a, and DAB2 in human THP-1 macrophages (Fig. 5E, F, G). This suggests that miR-99a-5p inhibits M2 polarization, which is generally associated with anti-inflammatory and tissue repair functions. In contrast, the expression of M1-related genes, including Traf1, IL-12a, and CD86, was significantly upregulated, indicating a shift towards a proinflammatory M1 phenotype.

To validate the specificity of miR-99a-5p’s effects, we conducted knockdown experiments*.* By knocking down miR-99a-5p in rat KCs and human THP-1 macrophages using anti-miR-99a-5p, we observed the opposite effects: the expression of M2-related genes increased, while the expression of the M1-related genes decreased (Figure S7). These results further support the role of miR-99a-5p in promoting M1 polarization and inhibiting M2 polarization.

We also performed cytokine array analyses (Fig. 5D and H) on the culture supernatant from miR-99a-5p mimic treated cells to measure the changes of inflammatory factors. The findings confirmed that miR-99a-5p promotes M1 polarization of macrophages by increasing the release of proinflammatory factors (IL-1α, IL-1β, IL-2, IL-6, IL-17 A, and TNF-α) while decreasing anti-inflammatory factors(IL-4 and IL-10)(Fig. 5D and H), further validating its role in skewing macrophage polarization toward M1*.*

**Fig. 6:**
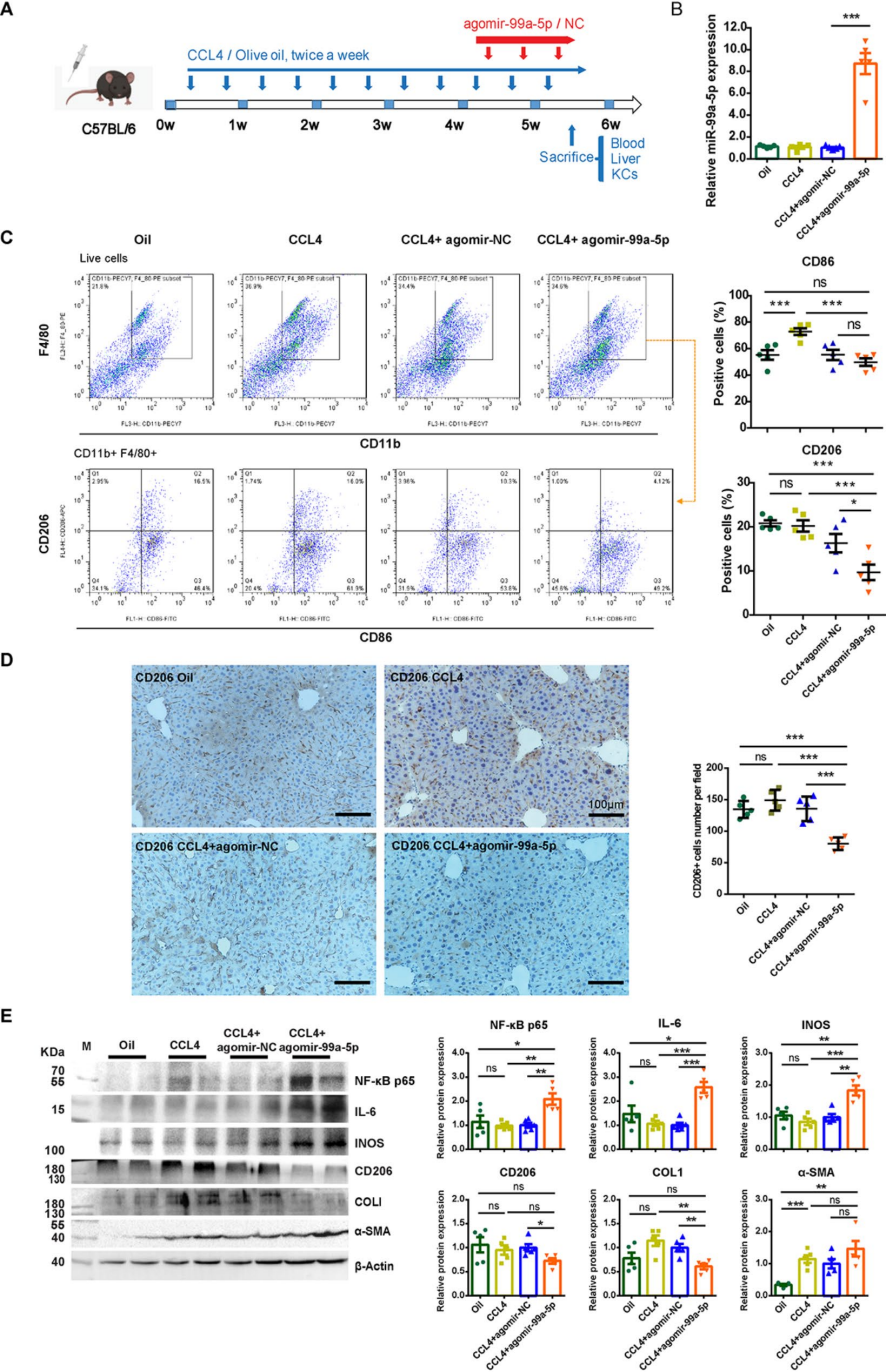
The effects of short-term activated HSC-sEV-specific miR-99a-5p on liver macrophage (KC) differentiation, inflammation and collagen deposition in a chronic liver injury mouse model. (**A**) Schematic representation of agomir-99a-5p treatment in a CCL4-induced chronic liver injury mouse model. (**B**) The expression of miR-99a-5p in KCs was determined by RT-qPCR and normalized to that of U6snRNA and to the miRNA agomir negative control (agomir-NC). (**C**) Surface marker expression in KCs gated on CD11b + F4/80 + primary liver mononuclear cells were determined by flow cytometry. Representative flow cytometry images and statistical histograms for CD86- and CD206-positive cells are provided. (**D**) Immunohistochemical staining of CD206 in liver tissue sections from each group. Representative images are provided, and positively stained cells are dark brown, scale bar = 100 μm. (**E**) The expression of NF-κB p65, IL-6, INOS, CD206, COLI, and α-SMA in the liver was detected by western blotting. β-Actin served as a loading control and was normalized to the mimic-NC treated group. Data from at least five mice for each group are presented as the mean ± SEM. Statistical significance was determined by one-way ANOVA, ***p < 0.001, **p < 0.01, *p < 0.05, ns, not significant

To explore the in vivo relevance of miR-99a-5p, we established a CCl4-induced liver injury mouse model and obtained in vivo activated HSCs (Figure S8A, C). We confirmed that the content of miR-99a-5p was also reduced in sEVs released by in vivo activated HSCs (Figure S8B, D). We then introduced agomir-99a-5p (a chemically modified miR-99a-5p agonist) intravenously into the mice with chronic liver injury* (*Fig. 6A*). *At the dosages used, no significant liver damage was observed (Figure S9A), ensuring that the treatment was safe. To track the delivery and uptake of agomir, we used 5-Cy3-labeled agomir-NC and observed that the agomir was efficiently taken up by liver macrophages as early as five minutes post-injection (Figure S9B). Treatment with agomir-99a-5p significantly increased the expression of miR-99a-5p in liver mononuclear cells* (*Fig. 6B*) *and reduced the percentage of CD206 + cells (p < 0.05) as detected by flow cytometry* (*Fig. 6C*) *and immunohistochemical staining* (*Fig. 6D*). *Conversely, treatment with antagomir-99a-5p, a miR-99a-5p inhibitor, reduced M1 markers and increased M2 markers (Figure S10A-F), confirming the impact of miR-99a-5p on macrophages polarization in vivo.

Finally, we examined the expression of proinflammatory regulators in the liver lysates from agomir-99a-5p-treated mice using western blotting. The results revealed that agomir-99a-5p increased the expression of NF-κB, IL-6 and iNOS, key regulators of inflammation. Additionally, the expression of COLI, a marker of fibrosis, was significantly decreased in the liver of agomir-99a-5p-treated mice (Fig. 6E) and confirmed by Sirius Red staining (Figure S9C). The expression of α-SMA, a marker of activated HSCs, was not significantly affected (Fig. 6E). On the contrary, antagomir-99a-5p increased collagen deposition (Figure S10G). These findings suggest that miR-99a-5p contributes to a proinflammatory microenvironment while reducing collagen deposition, a hallmark of liver fibrosis.

### Short-term activated HSC-sEV-specific miR-99a-5p-educated macrophages suppressed tumor progression

**Fig. 7:**
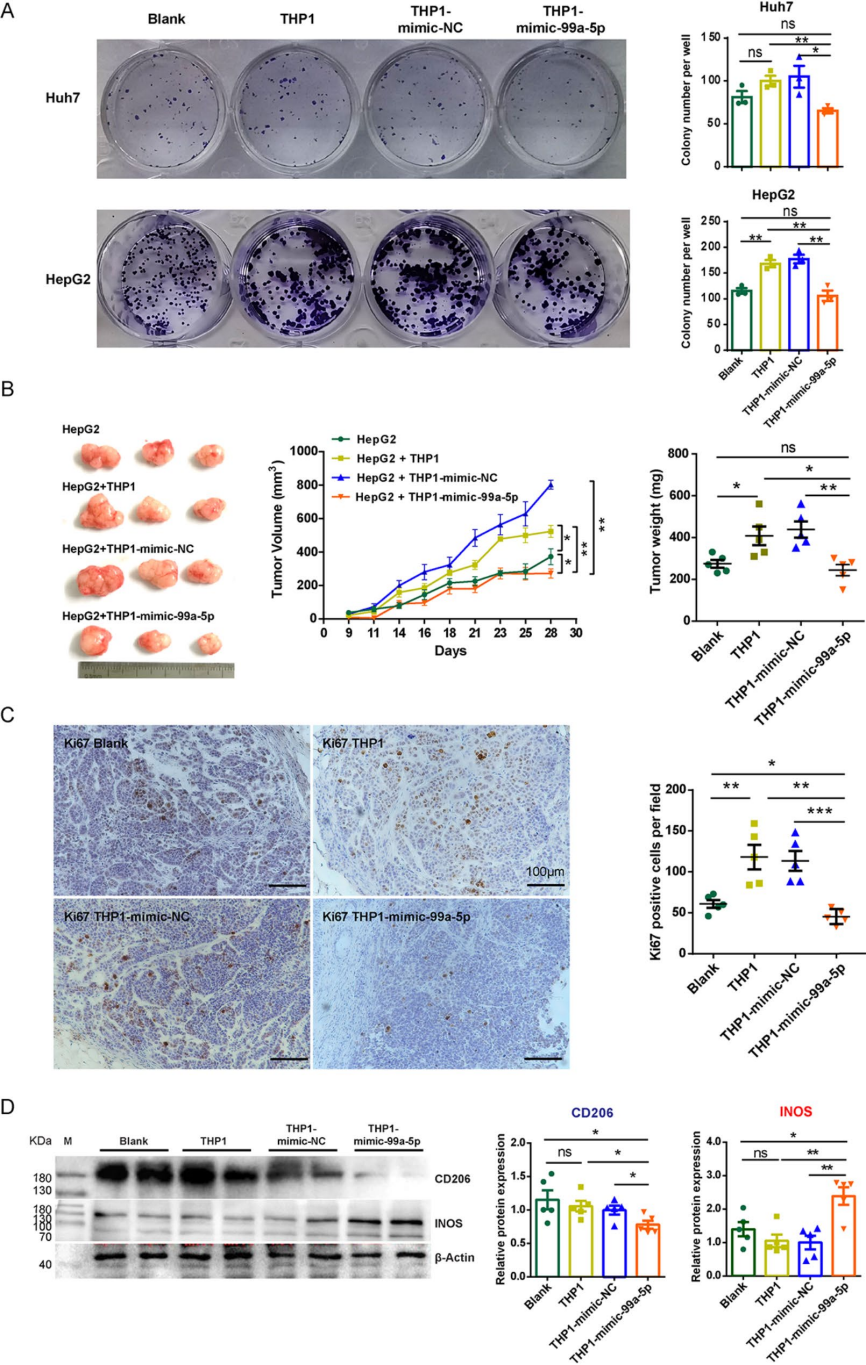
Short-term activated HSC-sEV-specific miR-99a-5p-educated macrophages suppressed the growth of hepatocellular carcinoma. THP-1 macrophages were pretreated with miR-99a-5p mimic or mimic negative control (mimic-NC) for 8 h. (**A**) Colony formation of Huh7 or HepG2 cells cocultured with miR-99a-5p mimic-pretreated THP-1 macrophages at a ratio of 5:1. (**B**) Nude mouse xenograft experiments. HepG2 cells were mixed and subcutaneously cotransplanted with miR-99a-5p mimic-pretreated THP-1 macrophages. The size of the tumor was measured at the indicated time points. The weight of the tumor was obtained on Day 28. (**C**) Immunohistochemical staining of Ki67 in paraffin sections of xenografts, scale bar = 100 μm. (**D**) The expression of CD206 and INOS in xenografts was detected by western blotting, β-Actin served as a loading control. For (**A**-**D**), the experiments performed with THP-1 macrophages pretreated with miRNA, mimic-NC served as a negative control. Representative images are provided. Data from at least three wells (**A**) or five mice (**B**-**D**) for each group were shown as the mean ± SEM. Statistical significance was determined by one-way ANOVA, ***p < 0.001, **p < 0.01, *p < 0.05, ns, not significant

To examine whether macrophages educated by miR-99a-5p could suppress HCC progression, we conducted both in vitro and in vivo experiments. First, we examined the effect of miR-99a-5p-educated macrophages on HCC cell growth in vitro. Human hepatoma cells (Huh7 and HepG2) were cocultured with human THP-1 macrophages that had been pre-treated with miR-99a-5p mimic. While macrophages generally promoted colony formation of Huh7 and HepG2 cells, the presence of miR-99a-5p mimic-treated macrophages significantly reduced the formation of clones (Fig. 7A). Next, we extended these findings in vivo by co-transplanting HepG2 cells with miR-99a-5p mimic-treated macrophages into nude mice. Tumors derived from this co-implantation exhibited reduced growth, as evidenced by smaller tumor sizes and lower tumor weights compared to controls (Fig. 7B). Furthermore, immunohistochemical analysis of Ki67, a proliferation marker, revealed decreased tumor cell proliferation, in the miR-99a-5p mimic-treated macrophage group (Fig. 7C).

Western blot analysis demonstrated that cotransplantion with miR-99a-5p mimic-treated macrophages led to a reduction in CD206, a marker of M2-like immunosuppressive macrophages, x`while increasing the expression of proinflammatory INOS, indicative of an M1-like immune-activated phenotype (Fig. 7D). These results align with our previous observations in the mouse liver fibrosis model, suggesting that miR-99a-5p shifts macrophages toward a proinflammatory, tumor-suppressive state*.*

*Conversely*, macrophages educated with an anti-miR-99a-5p inhibitor exhibited the opposite effect, enhancing the colony-forming ability of Huh7 and HepG2 cells in vitro (Figure S11A) and promoting HepG2 xenografts growth in vivo (Figure S11B, C). Taken together, these findings suggest that short-term activated HSC-sEV-specific miR-99a-5p reprograms macrophages toward an immune-activated state that suppresses HCC progression.

### CD93 is a potential target involved in miR-99a-5p induced macrophage differentiation

To explore the molecular mechanisms underlying miR-99a-5p-mediated macrophage differentiation, we aimed to identify potential target genes of miR-99a-5p that could regulate macrophage polarization*.* Using miRNA target prediction analyses, we cross-referenced differentially expressed mRNAs in 3dHSC-sEV-treated KCs with predicted miR-99a-5p targets from miRPathDB V2.0* (*https://mpd.bioinf.uni-sb.de/), TargetScan (https://www.targetscan.org/) and miRWalk (http://mirwalk.umm.uni-heidelberg.de/). CD93 emerged as a promising candidate for further validation, as it was among the top 20 genes significantly downregulated in 3dHSC-sEV-treated KCs (Table S4).

**Fig. 8:**
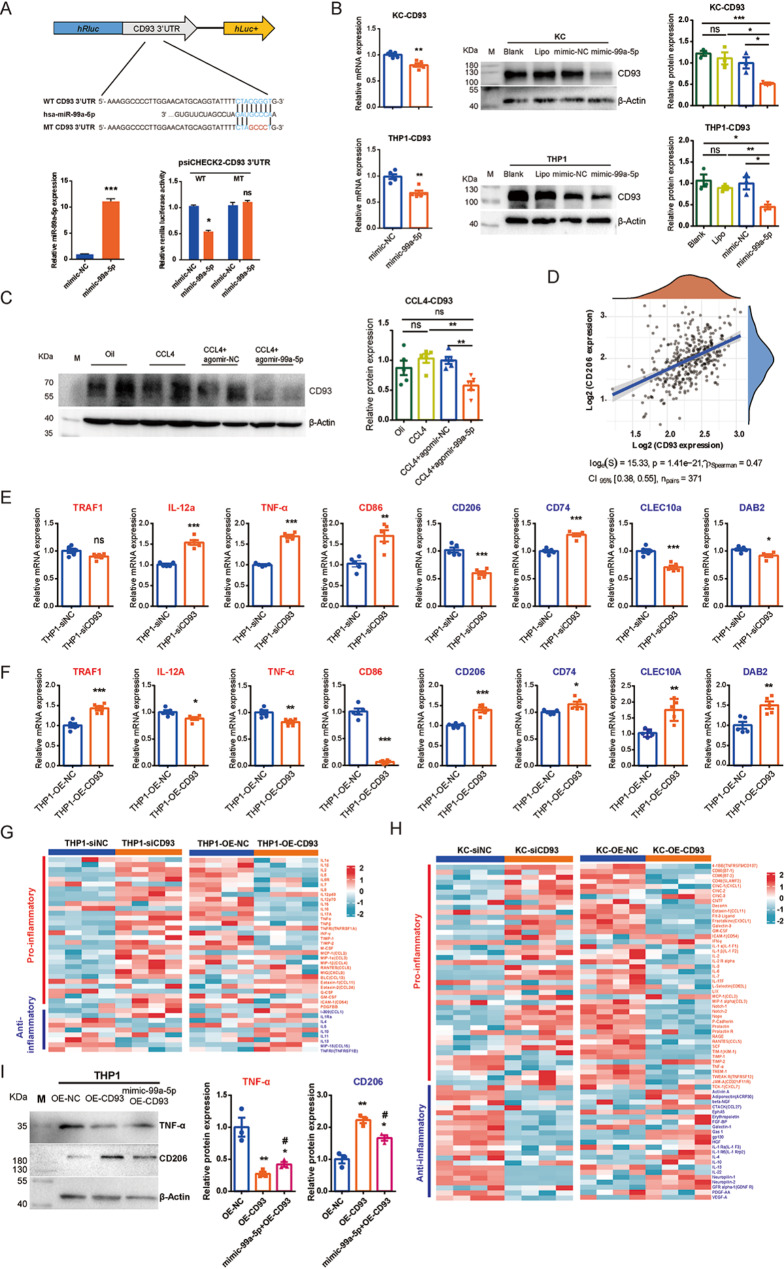
Short-term activated HSC-sEV-specific miR-99a-5p might influence macrophage differentiation by targeting CD93. (**A**) Dual-luciferase assay for the interaction between miR-99a-5p and the 3’UTR of CD93 mRNA in 293T cells. (**B**) The effects of the miR-99a-5p mimic on CD93 expression in primary rat liver macrophages (KCs) and human THP-1 macrophages were measured by RT-qPCR and western blotting. (**C**) The effects of agomir-99a-5p on CD93 expression in CCl4-induced chronic liver injury mice were detected by western blotting. (**D**) Spearman correlation between the expression of CD93 and CD206 in human HCC tissues; correlation *p* value = 1.41e-21, correlation coefficient = 0.47. (**E**) The mRNA expression of genes associated with HSC-sEV-induced macrophage differentiation in siCD93 treated human THP-1 macrophages was determined by RT-qPCR. (**F**) The mRNA expression of genes associated with HSC-sEV-induced macrophage differentiation in OE-CD93 treated human THP-1 macrophages was determined by RT-qPCR. (**G**, **H**) Cytokine array analyses for culture media from human THP-1 macrophages (**G**) or primary KCs (**H**) treated by siCD93, OE-CD93 and corresponding NC. Inflammatory factors are listed on the right side. Each row represents an individual inflammatory factor, and each column represents an individual sample. The scale represents normalized log2 inflammatory factor expression levels. Red font, proinflammatory factors; green font; anti-inflammatory factors. (**I**) The protein expression of TNF-α and CD206 in miR-99a-5p mimic treated CD93 overexpression human THP-1 macrophages were detected by western blotting. The relative protein expression was normalized to β-actin, and then to OE-NC. Statistical significance was determined by Student’s t test. Compared to OE-NC, ***p < 0.001, **p < 0.01, *p < 0.05; compared to OE-CD93, #*p* < 0.05. WT, wild type; MT, mutated type; NC, negative control

To confirm that miR-99a-5p directly regulates CD93 expression, we performed a dual-luciferase reporter assay, demonstrating that the miR-99-5p mimic significantly suppressed Renilla luciferase activity through the miR-99a-5p binding site in the 3’UTR of CD93 mRNA (Fig. 8A).

Next, we evaluated the regulatory effects of miR-99a-5p on CD93 expression both in vitro and in vivo. RT-qPCR and western blot analysis showed that miR-99a-5p mimic treatment significantly downregulated CD93 expression in both primary rat KCs and human THP-1 macrophages (Fig. 8B). Furthermore, in a CCl4-induced chronic liver injury mouse modle, administration of agomir-99a-5p similarly reduced the expression of CD93, as shown by western blot analysis (Fig. 8C).

To investigate the functional role of CD93 in macrophage differentiation, we analyzed RNA-Seq data from TCGA-LIHC (371 HCC tissue samples) and observed a positive correlation between CD93 expression and the M2 macrophage marker CD206 (Fig. 8D). This correlation suggested a potential role for CD93 in promoting an immunosuppressive M2 macrophage phenotype*.*

To functionally validate this observation, we knocked down CD93 in THP-1 macrophages using siRNA (Figure S12A) and analyzed the expression of macrophage polarization markers. CD93 knockdown reduced M2-related genes (CD206, CLEC10A, and DAB2) while increasing M1-related genes (TNF-α, IL-12a, and CD86), as detected by RT-qPCR (Fig. 8E). Conversely, overexpression of CD93 in THP-1 macrophages (Figure S12B) promoted M2 polarization by upregulating CD206, CLEC10A, and DAB2 while suppressing M1 markers TNF-α, IL-12a and CD86 (Fig. 8F).

To further confirm CD93’s role in macrophage-mediated immune regulation, we performed cytokine array analyses on the culture supernatant from THP-1 macrophages and primary rat KCs with either CD93 knockdown or overexpression (Figure S12), and further supported CD93’s anti-inflammatory effects (Fig. 8G and H). These analyses revealed that that CD93 overexpression suppressed proinflammatory cytokines (IL-1α, IL-1β, IL-2, IL-6, IL-17 A, and TNF-α), while increasing the release of anti-inflammatory cytokines (IL-4 and IL-10). Conversely, CD93 knockdown exhibited the opposite effects, reinforcing its role in promoting an anti-inflammatory macrophage phenotype (Fig. 8G, H).

Finally, we investigated whether miR-99a-5p regulates macrophage polarization via CD93*.* Transfection of miR-99a-5p mimics counteracted the effects of CD93 overexpression (Figure S13A), reversing the upregulation of M2-associated gene and downregulation of M1-related genes as confirmed by RT-qPCR (Figure S13B) and western blotting (Fig. 8I).

Our findings indicate that miR-99a-5p suppresses CD93 expression, thereby shifting macrophages from an anti-inflammatory M2 phenotype to a proinflammatory M1 phenotype. This regulation suggests that miR-99a-5p contributes to an immune-activated tumor microenvironment, potentially influencing HCC progression through macrophage polarization.

## Discussion

In this study, we redefined the in vitro culture activation model of primary HSCs to mimic the in vivo activation process of HSCs. Short-term activated HSCs (3dHSCs) resemble the HSCs in the early stage of liver injury, while long-term activated HSCs (14dHSCs) represent those associated with chronic liver injury. We further revealed that the short-term activated HSCs reduced the M2-like gene expression in KCs and activated KCs to the M1-like state. In contrast, the long-term activated HSCs lost the ability to induce the proinflammatory phenotype of KCs and instead induced M2-like differentiation of MOs, which is consistent with the immune suppressive role of activated HSCs on monocytes and in chronically injured cirrhotic liver [8]. We identified the differential miRNAs carried by 3dHSC- and 14dHSC-sEVs. The proinflammatory effect of short-term activated HSC-sEV-specific miR-99a-5p was verified, and the underlying mechanism was explored.

To evaluate the impacts of HSCs at different activation stages on the liver macrophage, we cocultured primary KCs and MOs with short-term and long-term activated HSC-CMs or sEVs. We were surprised to observe that 3dHSC-CMs and sEVs cocultures increased the adhesion of KCs and MOs with more spread-out spindle-like morphology. Daphne et al. reported that macrophages activated with proinflammatory IFN-γ/LPS were predominantly spindle-like (M1), and anti-inflammatory activation induced a circular morphology (M2) [40]. We proposed that 3dHSC-CM and sEV might induce M1 activation of macrophage. Consistent with morphological changes, 3dHSC-sEVs treatment increased M1-related gene expression in KCs, while 14dHSC-sEVs treatment increased M2-related gene expression in MOs. Moreover, 14dHSC-sEV-treated KCs showed a similar gene expression pattern as untreated KCs. It was encouraging that the effects of long-term activated rat HSC-sEVs on MOs were consistent with our previous finding that LX2 (a human cell line resembling activated stellate cells) educated human MOs to an M2-like phenotype [8]. The more important finding of the present study is that the sEVs from short-term activated HSCs were able to activate KCs to M1-like cells, revealing a proinflammatory effect of short-term activated HSCs. Long-term activated HSCs lost the ability to activate KCs to the M1 state. Therefore 14dHSC-sEVs treated KCs maintained M2-like differentiation [27]. We demonstrated that HSCs at differential activation stages have different impacts on macrophage polarization. As for healthy individuals, the quiescent HSCs will be activated upon liver injury, and sEVs released by these short-term activated HSCs can promote M1 polarization of macrophages and induce a proinflammatory microenvironment. However, in individuals with chronic liver injury, the HSCs are continuously activated. The sEVs released by these long-term activated HSCs could no longer activate macrophages to M1-like status and even induce M2-like differentiation, contributing to an immunosuppressive microenvironment.

In this study, an important finding is that short-term activated HSC-sEVs induce M1-like differentiation in KCs; in contrast, the induction of M2-like differentiation by long-term activated HSC-sEVs is more effective in MOs. In the liver, the recruited monocytes (MOs) are critical players during the pathogenic challenge; they differentiate in M1-like phenotype and initiate the inflammatory response [26], whereas resident macrophages (KCs) in M2-like mode have essential roles in tissue homeostasis and the resolution of inflammation, they can also promote tumor growth [27]. The differential effects of HSC-sEVs on these two cell types may be caused by their respective properties. When the HSC-sEV-treated KCs/MOs acquired the opposite phenotype, the effects detected would be more pronounced.

To elucidate the molecular mechanism underlying the effects of HSC-sEVs on macrophages, we performed IPA analyses with the differentially expressed gene expressed genes in 3dHSC- and 14dHSC-sEVs treated KCs. IPA analyses of differentially expressed genes in 3dHSC-sEVs treated KCs revealed that the activated pathways were strongly associated with TNF signaling and infection response* (*Fig. 2G*). *Upstream regulator analysis *(*Fig. 2H*) *predicted activation of NF-κB and key M1-associated factors such as TNF and IL1B, which are well known to trigger NF-κB activation via receptor-mediated recruitment of the IKK complex, leading to phosphorylation and nuclear translocation of NF-κB p65. This finding strongly suggests that short-term activated HSC-sEVs treatment in KCs primarily activates the canonical TNF/NF-κB pathway. In contrast, 14dHSC-sEV-treated KCs indicated activation of M2-associated regulators (e.g., IL-10, IL-6), which are known to signal predominantly via the STAT3 pathway and promote anti-inflammatory responses. Thus, the temporal differences in HSC-sEV cargo appear to dictate distinct intracellular responses: short-term activated HSC-sEVs treatment predominantly triggers pro-inflammatory, NF-κB–dependent signaling, while long-term activated HSC-sEVs treatment might shift the balance toward anti-inflammatory, STAT3-mediated signals. Additional experiments are needed to further dissect these pathways.

To explore the molecules in sEV that affect macrophage differentiation, we focused on the sEV-miRNAs, which are essential cargoes of sEVs packaged from parent cells [15]. Although it has been reported that the absolute quantity of miRNAs per individual EV is generally low [41], the biological significance of miRNAs in sEV-mediated intercellular communication likely depends on both the selective packaging mechanisms and the cumulative effect of multiple sEVs delivering their miRNA cargo to target cells [42, 43]. In the present study, the top 20 miRNAs account for about 84-85% of the reads of all the sEV-miRNAs detected (Table S9)*.* Small RNA sequencing showed that the HSC-sEVs contained fewer detectable miRNA species than parent HSCs. Among the 48 different miRNAs in cells and the 52 different miRNAs in sEVs, only 12 (≤ 25%) overlapped, of which 11 showed the same variation trend. Although it is believed that sEV-miRNAs represent the state of parent cells [12], our data suggest that the changes of miRNA components in cells and corresponding vesicles under pathological conditions are not necessarily consistent. This inconsistency might be caused by the different subcellular distributions of miRNAs. Moreover, the miRNAs packaged into sEVs could be sorted or even enriched [39]. This study focused on the 11 miRNAs that showed the same variation trend in both parent HSCs and sEVs. In this way, we can ensure that when we identify the target miRNA and intervene, the parent and surrounding cells receive the same and appropriate regulatory information and function as a whole. Among the 11 miRNAs, the expression of miR-221-3p has been reported to be increased in activated HSCs and to promote liver fibrosis [44]. The role of miRNA-99a-5p and miR-139-5p has not been reported in HSCs but in some malignant tumors, including HCC, breast cancer, colorectal cancer, and bladder cancer. They could act as tumor suppressors and have diagnostic and prognostic value [45, 46, 47]. The miRNAs associated with better prognosis of HCC, such as miR-99a-5p and miR-139-5p, were lost in long-term activated HSC-sEVs, while the cancer-promoting miR-221-3p increased. These findings are consistent with previous studies about the role of long-term activated HSCs in promoting HCC progression and move one step forward to suggest a protective role of short-term activated HSC-sEV-specific miRNAs in liver cancer.

When investigating the biological relevance of the short-term activated HSC-sEV-specific miRNAs on macrophage differentiation, we focused on miR-99a-5p, one of the most abundant and highly expressed miRNAs in 3dHSC-sEVs. The in vitro studies were carried out with rat KCs and THP-1 macrophages. Treatment with miR-99a-5p reduced M2 differentiation in primary rat KCs and human THP-1 macrophages. In vivo, in a CCL4-induced liver fibrosis mouse model, miR-99a-5p reduced M2 marker expression and simultaneously reduced the production of collagen type I and III. It has recently been reported that targeting profibrogenic M2 macrophages alleviates pulmonary fibrosis [48], and cytotherapy with M1-polarized macrophages ameliorates liver fibrosis [49]. Here, we found that sEVs and their specific miRNAs from short-term activated HSCs could induce a proinflammatory phenotype of macrophages, suppress M2 differentiation, and alleviate liver fibrosis. We further confirmed the tumor suppressive effects of the macrophages educated by short-term activated HSC-sEV-specific miRNA. In vitro, in a macrophage and tumor cell coculture system, miR-99a-5p-pretreated macrophages inhibited the colony formation ability of Huh7 and HepG2 cells. In vivo, in a macrophage and tumor cell cotransplantation model, miR-99a-5p-pretreated macrophages inhibited the growth of hepatoma xenografts with reduced CD206 and increased iNOS expression. Based on the above findings, we propose that in contrast to long-term activated HSCs, short-term activated HSCs could inhibit tumor progression by inducing proinflammatory macrophage differentiation. Supplementation of short-term activated HSC-sEV-specific miRNA may activate the liver immune microenvironment, providing a new adjuvant strategy for preventing and treating liver cancer.

Finally, we explored the potential mechanism involved in miR-99a-5p-induced immune activation. We focused on CD93, a predicted target of miR-99a-5p. CD93 is a C-type lectin transmembrane protein, and its cytoplasmic tail can interact with the actin cytoskeleton, facilitating its reorganization for functions such as phagocytosis, cellular adhesion, and migration [50]. It can enhance the phagocytosis in monocytes and macrophages and contribute to M2 differentiation [51]. More interestingly, Chen et al. recently reported that blockade of the CD93 pathway sensitized mouse tumors to immune checkpoint therapy [52]. In this study, we confirmed CD93 as a target of miR-99a-5p. The effect of CD93 on macrophage polarization was also examined. In human HCC tissues, the expression of CD93 was positively correlated with the M2-specific gene CD206. In KCs and THP-1 cells, knocking down of CD93 increased the expression of M1-specific genes and decreased the expression of M2-specific genes. At the same time, overexpression of CD93 had the opposite effects. Therefore, the short-term activated HSC-sEVs containing higher miR-99a-5p might reduce CD93 expression in macrophages and contribute to proinflammatory differentiation. In contrast, long-term activated HSC-sEVs lost miR-99a-5p, and the expression of CD93 increased in macrophages, which partially contributed to M2 differentiation. CD93 is known as complement component 1q receptor (C1qR). At the same time, C1q has been shown to induce a pro-resolving phenotype in macrophages, reducing pro-inflammatory cytokine production and increasing anti-inflammatory cytokine release [53]*. *CD93 may influence immune cell functions, potentially affecting macrophage polarization through interaction with the complement system. In subsequent studies, we will continue to explore the mechanism for CD93-regulated macrophage differentiation.

However, the compositions of sEV are diverse, and the present study only sampled miRNAs, and only a single miRNA was investigated. This study has inevitable limitations. Firstly, in the present study, we notice that the 3dHSC-sEVs increased the expression of all listed M1-related genes KCs *(*Fig. 2D*)*, but in Proteinase K-treated 3dHSC-sEVs co-cultured KCs, the expression of Mmp2 was reduced (Figure *S4*B*). *Interestingly, Mmp2 was also reduced in mimic-miR-99a-5p treated KCs* (*Fig. 5A*). *This observation suggests that the phenotypic effect transferred to the recipient cells partially requires EV-protein surface cargo. Secondarily, the biological role of miR-99a-5p was only validated by gain or loss function. We will continue to carry out the functional validation of this miRNA as sEV cargo. There are several unanswered questions that still need to be solved in future research.

## Conclusions

In conclusion, our findings suggest that HSCs at different activation stages affect macrophage differentiation differentially and provide a new perspective for the immune regulatory function of activated HSCs. Consistent with previous findings in the fibrotic livers with chronic injury, sEVs from long-term activated HSCs contributed to an immunosuppressive phenotype of macrophages. However, the sEVs released by short-term activated HSCs could activate proinflammatory macrophages, which suggested the role of HSCs in immune surveillance in the early stage of liver injury. We also identified sets of short-term and long-term activated HSC-sEV-specific miRNAs and validated the proinflammatory and tumor suppressive effects of one of the short-term activated HSC-sEV-specific miRNAs. Our study will help elucidate the role of HSCs in the liver microenvironment, define actionable therapeutic targets for liver fibrosis, and block the crosstalk among HSCs, immune cells, and tumor-initiating cells to establish effective strategies for early prevention and treatment of fibrosis-related liver cancer.

## Electronic supplementary material

Below is the link to the electronic supplementary material.

Supplementary Material 1

Supplementary Material 2

Supplementary Material 3

Supplementary Material 4

## Data Availability

The data generated during the current study are available from the corresponding author on reasonable request.
