# Supplementary material for: Reduction of alternative polarization of macrophages by short-term activated hepatic stellate cell-derived small extracellular vesicles
Source: J Exp Clin Cancer Res. 2025 Apr 10;44:117. doi: 10.1186/s13046-025-03380-0 (PMC11983935; doi:10.1186/s13046-025-03380-0)
Supplement: Supplementary file 1 — Supplementary Material 1 [file 13046_2025_3380_MOESM1_ESM.docx]

**Supplemental Information**

**Supplemental Figures and Figure Legends**


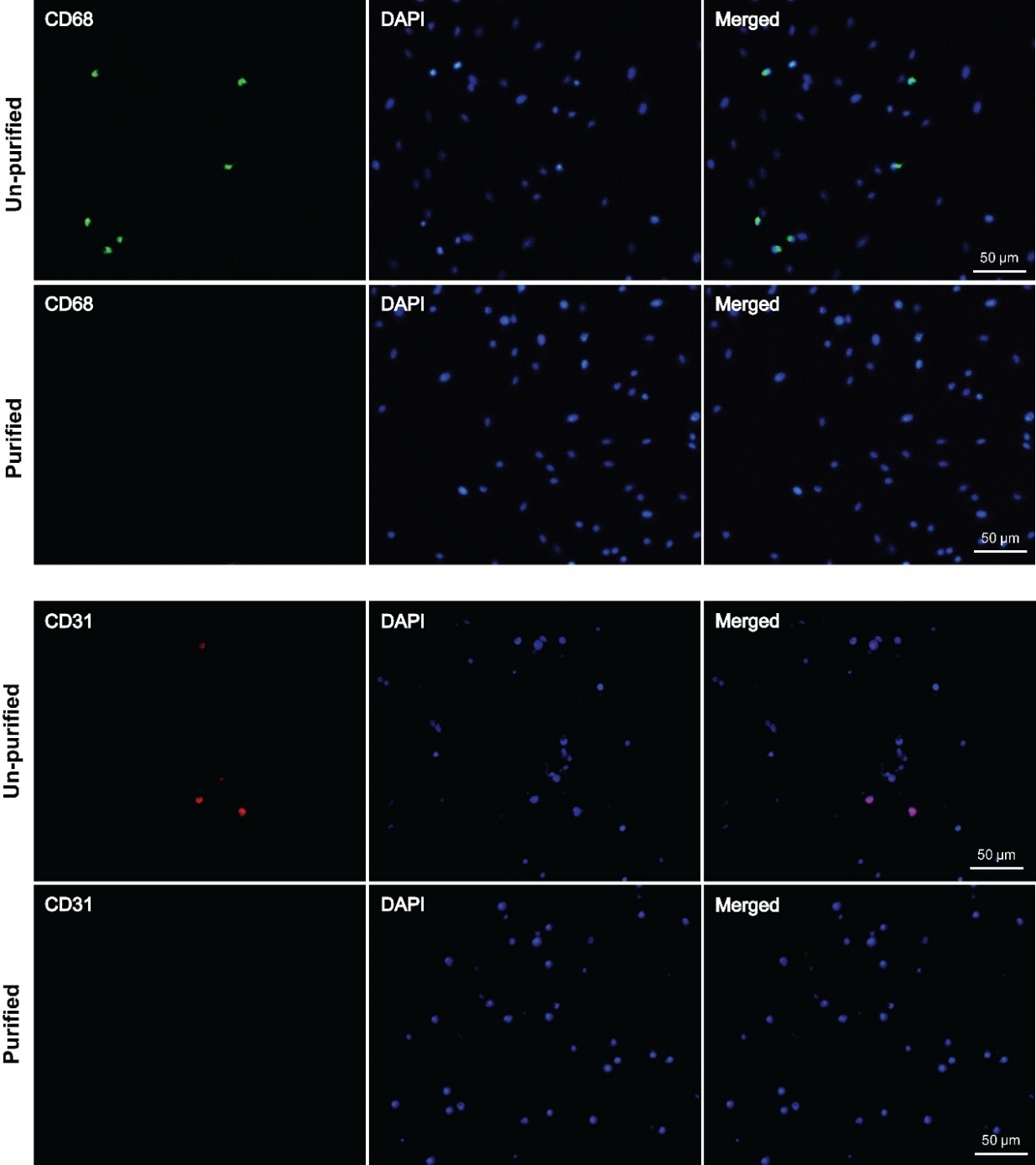


**Figure S1. MACS purification of primary rat HSCs.** MACS-purified primary rat HSCs cultured for 2 days were examined by immunofluorescence staining for the macrophage marker CD68 (green) and endothelial cell marker CD31 (red) to exclude potential contamination of macrophages and endothelial cells, scale bar = 50 μm. MACS, magnetic activated cell sorting.


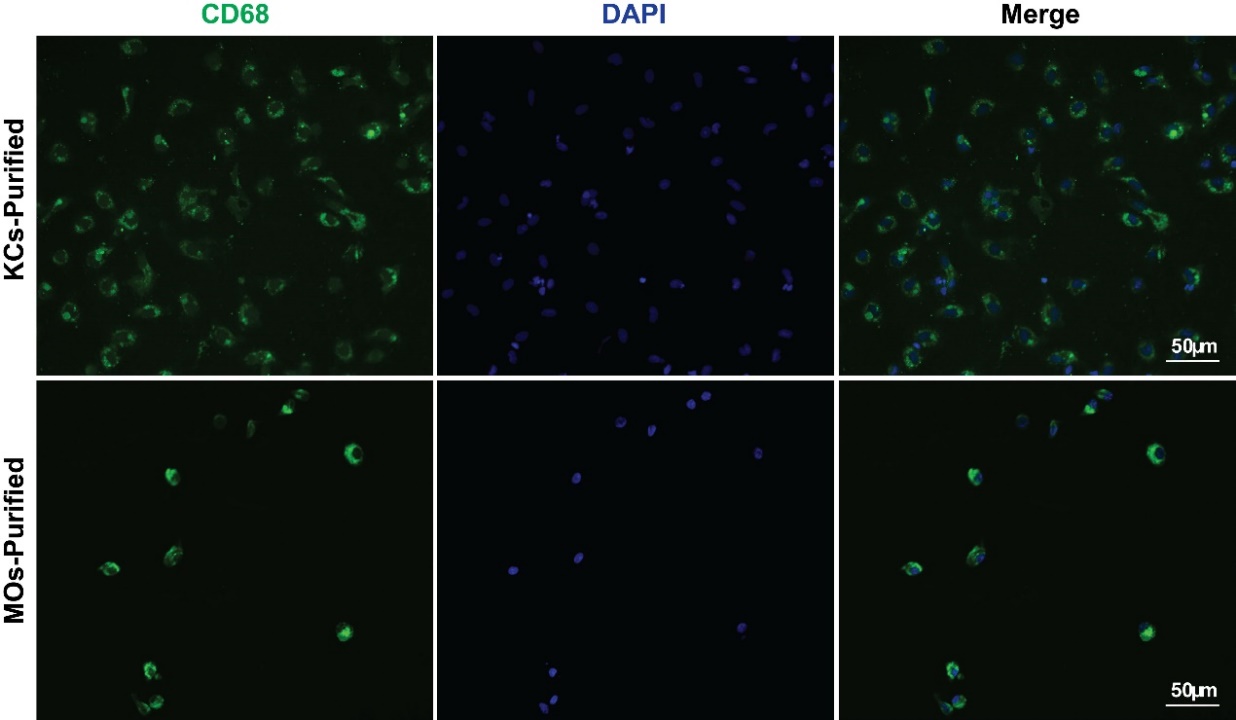


**Figure S2. MACS purification of primary rat liver resident macrophages (Kupffer cells, KCs) and bone marrow-derived monocytes (MOs).** Primary rat KCs and MOs were obtained by density gradient centrifugation combined with CD45 or CD11b/c MACS positive selection. MACS-purified primary rat KCs and MOs were examined by immunofluorescence staining for CD68 (ALEXA 488, green), and the cellular nuclei were stained with DAPI (blue), scale bar = 50 μm. MACS, magnetic activated cell sorting.


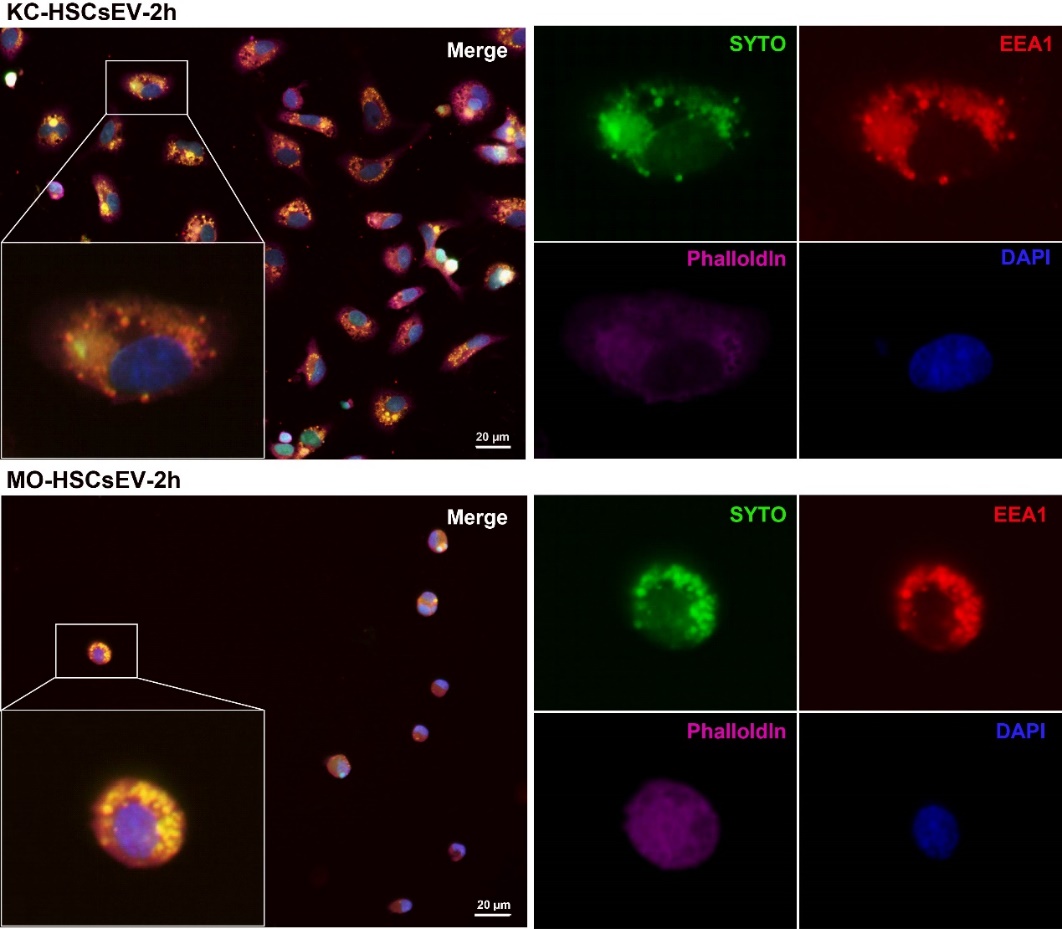


**Figure S3. Uptake of HSC-sEVs by primary rat liver resident macrophages (Kupffer cells, KCs) and bone marrow-derived monocytes (MOs).** Primary rat KCs and MOs were incubated with SYTO (green)-labeled primary HSC-sEVs for 2 hours. The early endosomes were stained with EEA1(red), and cytoskeleton was stained with phalloidin (violet), the cellular nuclei were counter stained with DAPI (blue), scale bar = 20 μm.


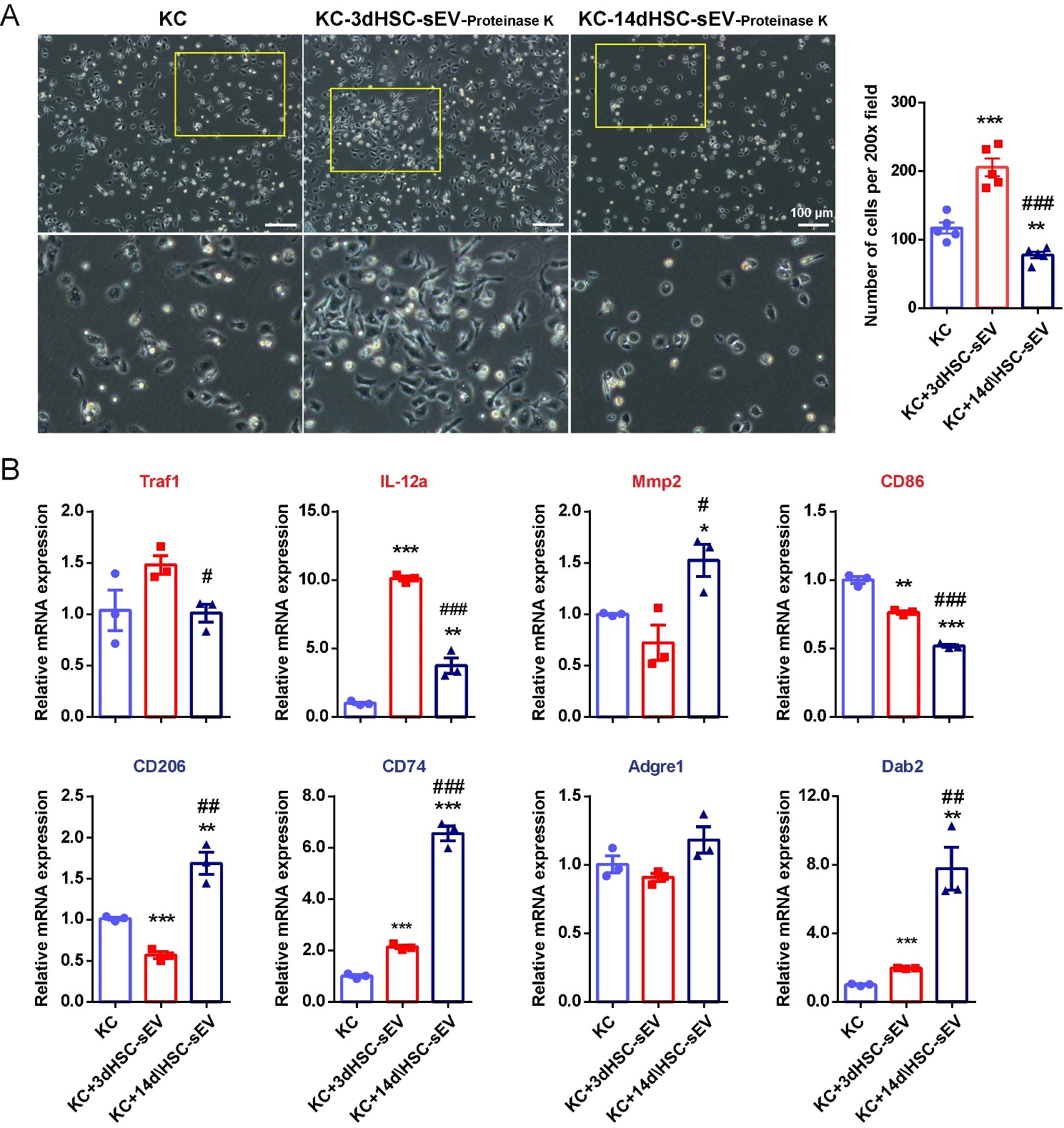


**Figure S4. The effects of short-term activated HSC (3dHSC)- and long-term activated HSC (14dHSC)-derived sEVs (treated with Proteinase K) on liver macrophage (KC).** (A) Morphology of HSC-sEV-cocultured KCs, scale bar = 100 μm. (B) The gene expression levels of key macrophage biomarkers in KCs cocultured with 3dHSC- or 14dHSC-sEVs treated with Protease K were determined by RT-qPCR. Statistical significance was determined by Student’s t test relative to untreated KCs, *** p < 0.001, ** p < 0.01, * p < 0.05; and Student’s t test relative to 3dHSC-sEV-cocultured KCs, ### p < 0.001, ## p < 0.01, # p < 0.05.


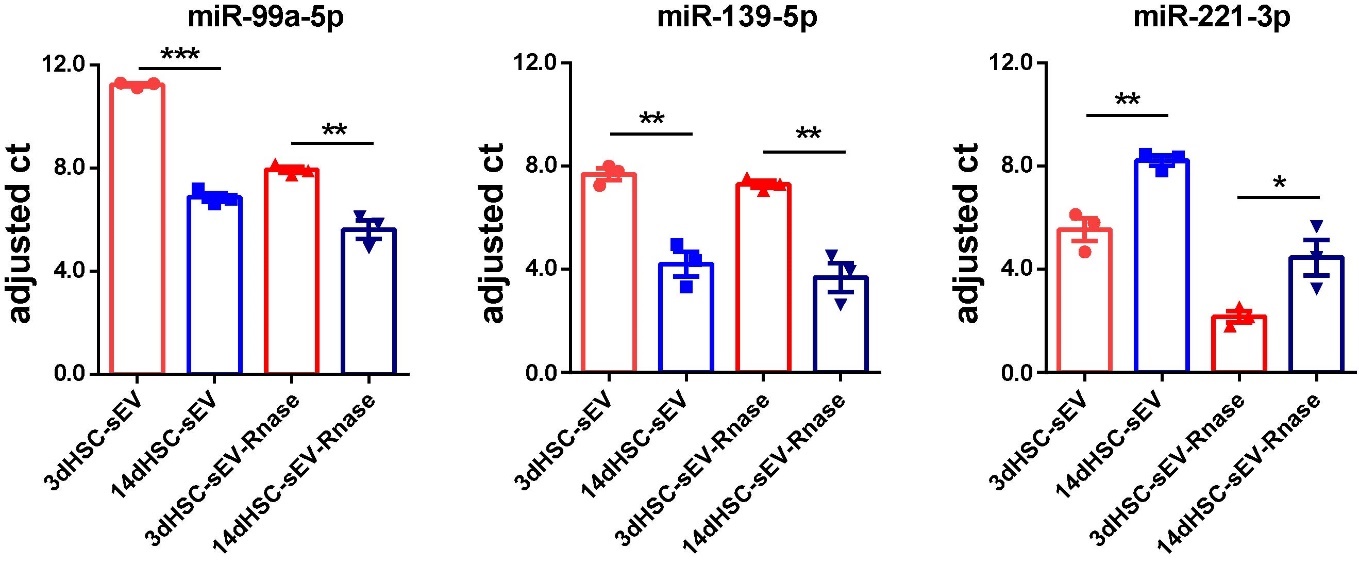


**Figure S5. The expression of miR-99a-5p, miR-139-5p, and miR-221-3p in untreated and RNase-treated sEVs from rat primary HSCs.** The relative expression of miRNA was expressed as adjusted Ct (40-Ct), and statistical significance was determined by Student’s t test, *** p < 0.001, ** p < 0.01, * p < 0.05, ns, not significant.


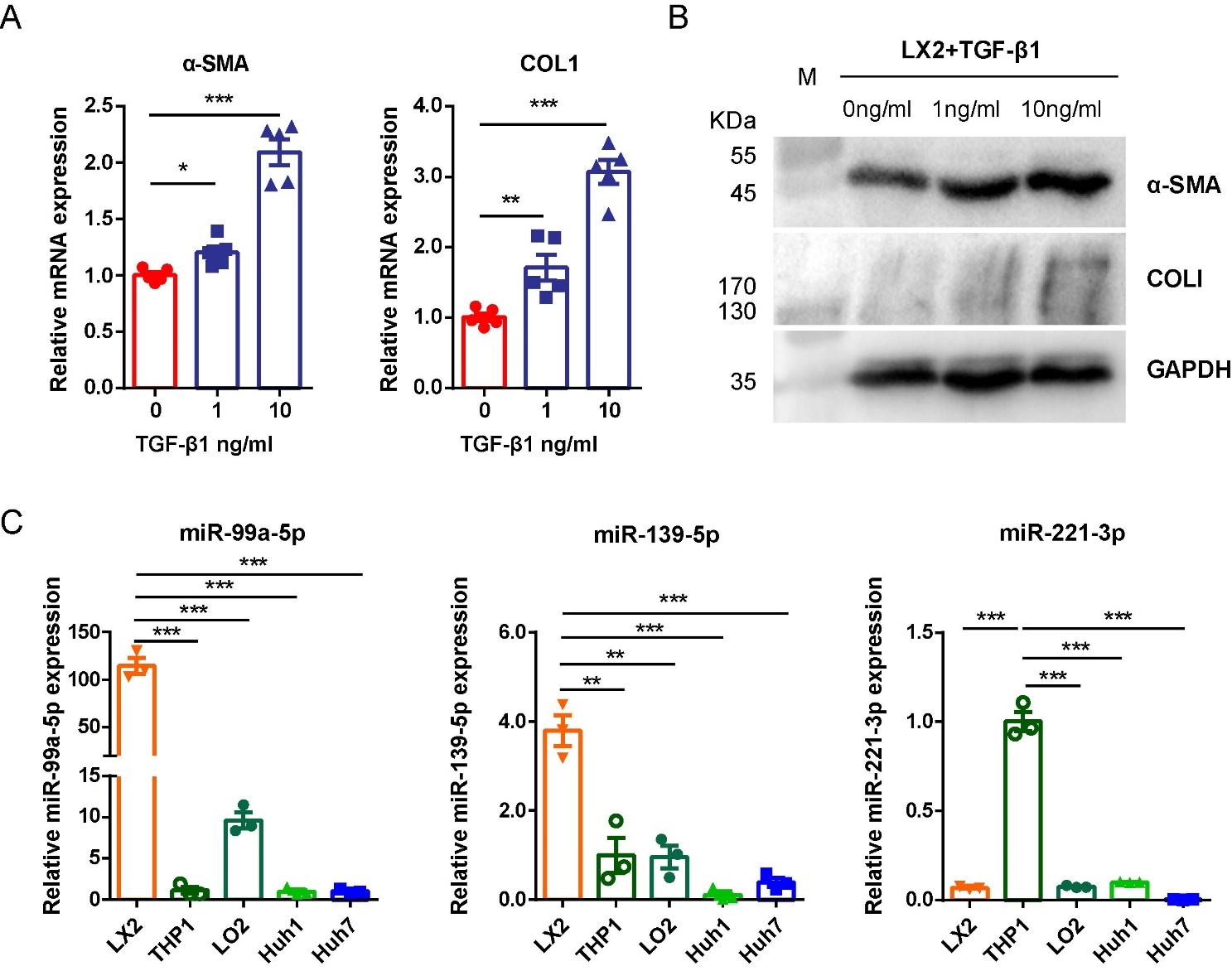


**Figure S6. Validation of TGF-β-induced activation of the human hepatic stellate cell line LX2.** **(**A) The mRNA expression of α-SMA and COLI in the TGF-β-treated human hepatic stellate cell line LX2 measured by qRT-PCR. Compared to untreated LX2, GAPDH served as a reference gene, and statistical significance was determined by Student’s t test. **(**B) The protein expression of α-SMA and COLI in the TGF-β-treated human hepatic stellate cell line LX2 detected by western blotting. GAPDH served as a loading control. (C) The expression of miR-99a-5p, miR-139-5p, and miR-221-3p in human hepatic stellate cell line LX2, PMA-differentiated human THP-1 macrophages, human liver cell line LO2, and hepatoma cell lines (Huh1 and Huh7) was determined by qRT-PCR. The relative miRNA expression was normalized to that of U6snRNA, and then to THP1 and are presented as the mean ± SEM from three independent tests. *** *p* < 0.001, ** *p* < 0.01, * *p* < 0.05.


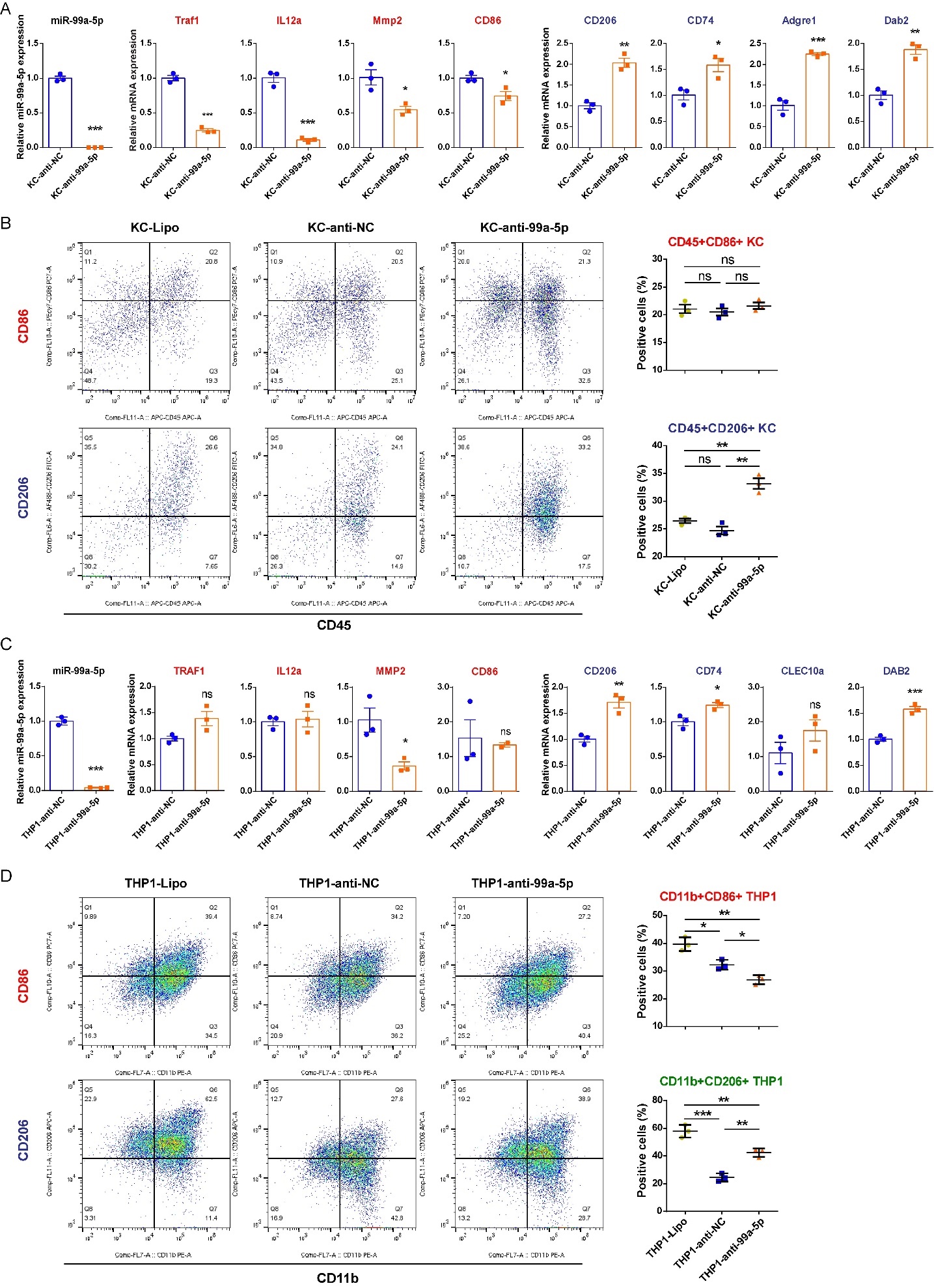


**Figure S7. The effects of anti-miR-99a-5p on primary rat liver macrophage (KC) and human THP-1 macrophage differentiation.** (A, C) The expression of miR-99a-5p and the mRNAs of 8 genes associated with HSC-sEV-induced macrophage differentiation in anti-miR-99a-5p-treated primary rat KCs (A) and human THP-1 macrophages (C) was determined by qRT-PCR. The relative miR-99a-5p expression was normalized to that of U6snRNA, and the relative gene expression was normalized to that of β-Actin and then to anti-miRNA negative control (anti-NC). Red font, highly expressed in 3dHSC-sEV cocultured KCs, green font, highly expressed in 14dHSC-sEV cocultured KCs. (B, D) Surface marker expression in anti-miR-99a-5p-treated primary rat KCs (B) and human THP-1 macrophages (D) was determined by flow cytometry. Representative flow cytometry images and statistical histograms for CD86- and CD206-positive cells in each group are provided. Data are presented as the mean ± SEM, and statistical significance was determined by Student’s t test, *** *p* < 0.001, ** *p* < 0.01, * *p* < 0.05, ns, not significant.


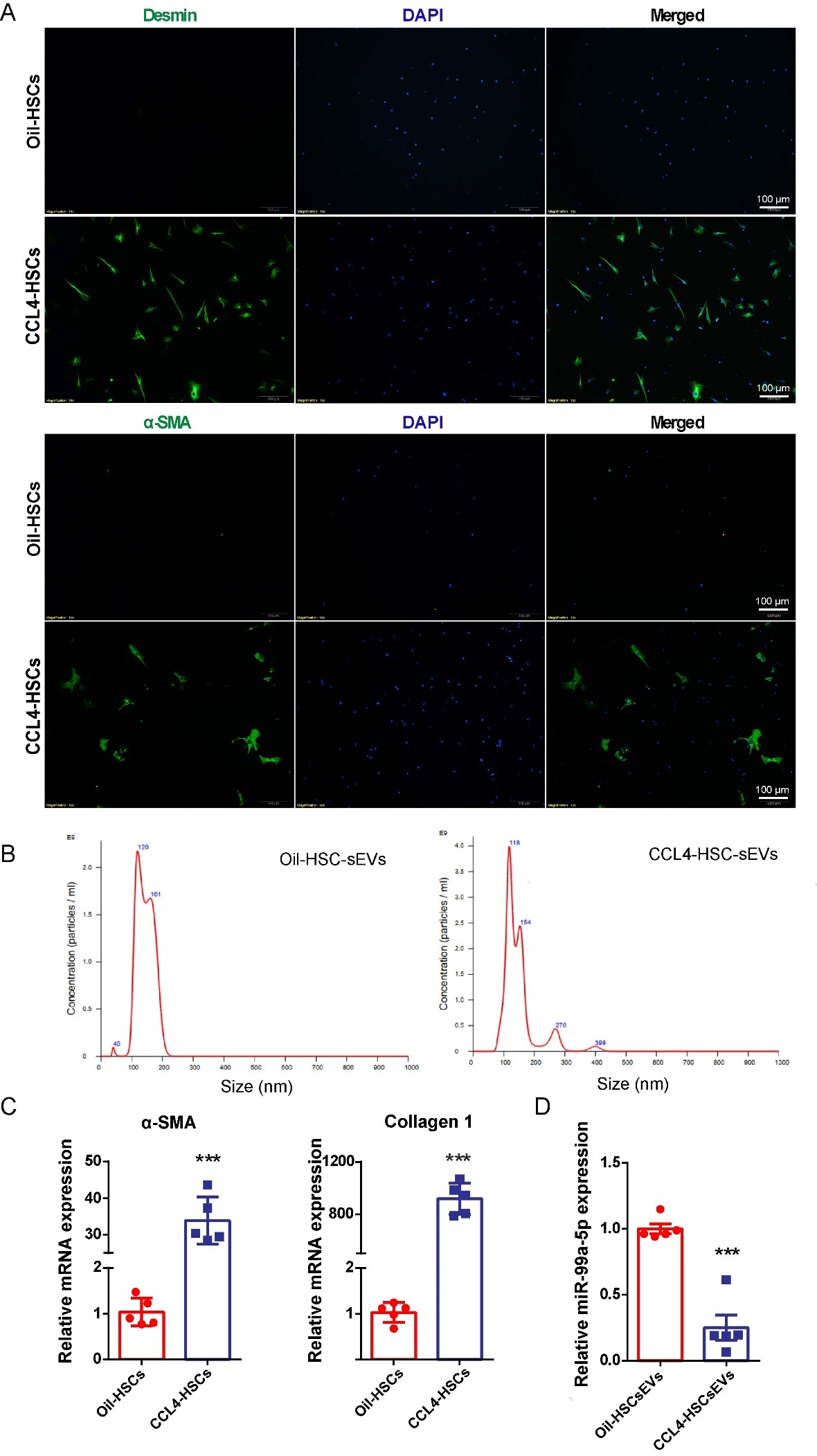


**Figure S8. Isolation and characterization of primary HSCs from** **mice with CCL4-induced chronic liver injury and detecting of miR-99a-5p loaded in corresponding sEVs.** (A) Immunofluorescence staining of desmin and α-smooth muscle actin (α-SMA) in primary HSCs isolated from mice with CCl4-induced chronic liver injury and oil-treated vehicle control, desmin and α-SMA (green); nuclei were counterstained with DAPI (blue), scale bar = 100 μm. (B) The representative size distribution of isolated particles and their concentrations were determined by nanoparticle tracking analyses. (C) The mRNA expression of HSC activation markers α-SMA and Collagen I in primary HSCs were determined by qRT-PCR. The relative mRNA expression was normalized to that of β-Actin and then to oil-treated control. (D) The expression of miR-99a-5p in sEVs released from primary HSCs was determined by qRT-PCR. The relative miR-99a-5p expression was normalized to that of U6snRNA and then to oil-treated control. Data are presented as the mean ± SEM, and statistical significance was determined by Student’s t test, *** *p* < 0.001.


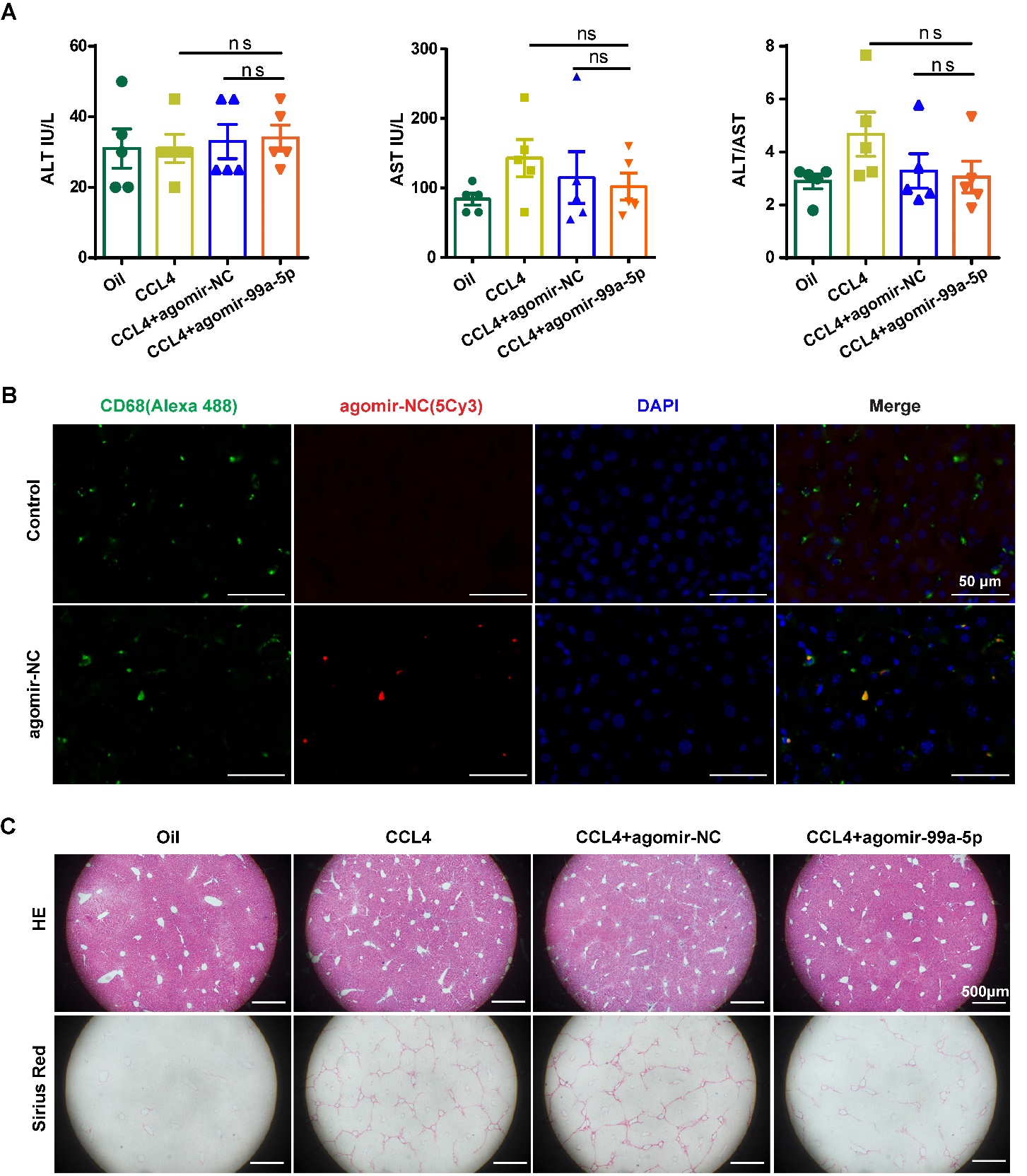


**Figure S9. The effects of short-term activated HSC-sEV-specific miR-99a-5p on KC differentiation, inflammation and collagen deposition in a chronic liver injury mouse model.** (A) Liver function tests of the agomir-99a-5p-treated chronic liver injury mouse model induced by CCL4. Serum alanine transaminase (ALT) and aspartate transaminase (AST) levels were measured 48 h after the last dose of agomir-99a-5p. Data from at least five mice for each group are presented as the mean ± SEM, and statistical significance was determined by Student’s t test, ns, not significant. (B) Uptake of agomir by KCs. KCs were labeled with immunofluorescence staining of CD68 (Alexa 488, green), agomir-NC was labeled with 5-Cy3 (red), and nuclei were counterstained with DAPI (blue) in frozen sections of mouse livers. Scale bar = 50 μm. (C) H&E and Sirius Red staining of liver sections from each group. Representative images from at least five mice for each group are provided. Scale bar = 500 μm. Data from at least five mice for each group are presented as the mean ± SEM, and statistical significance was determined by one-way ANOVA, ns, not significant.


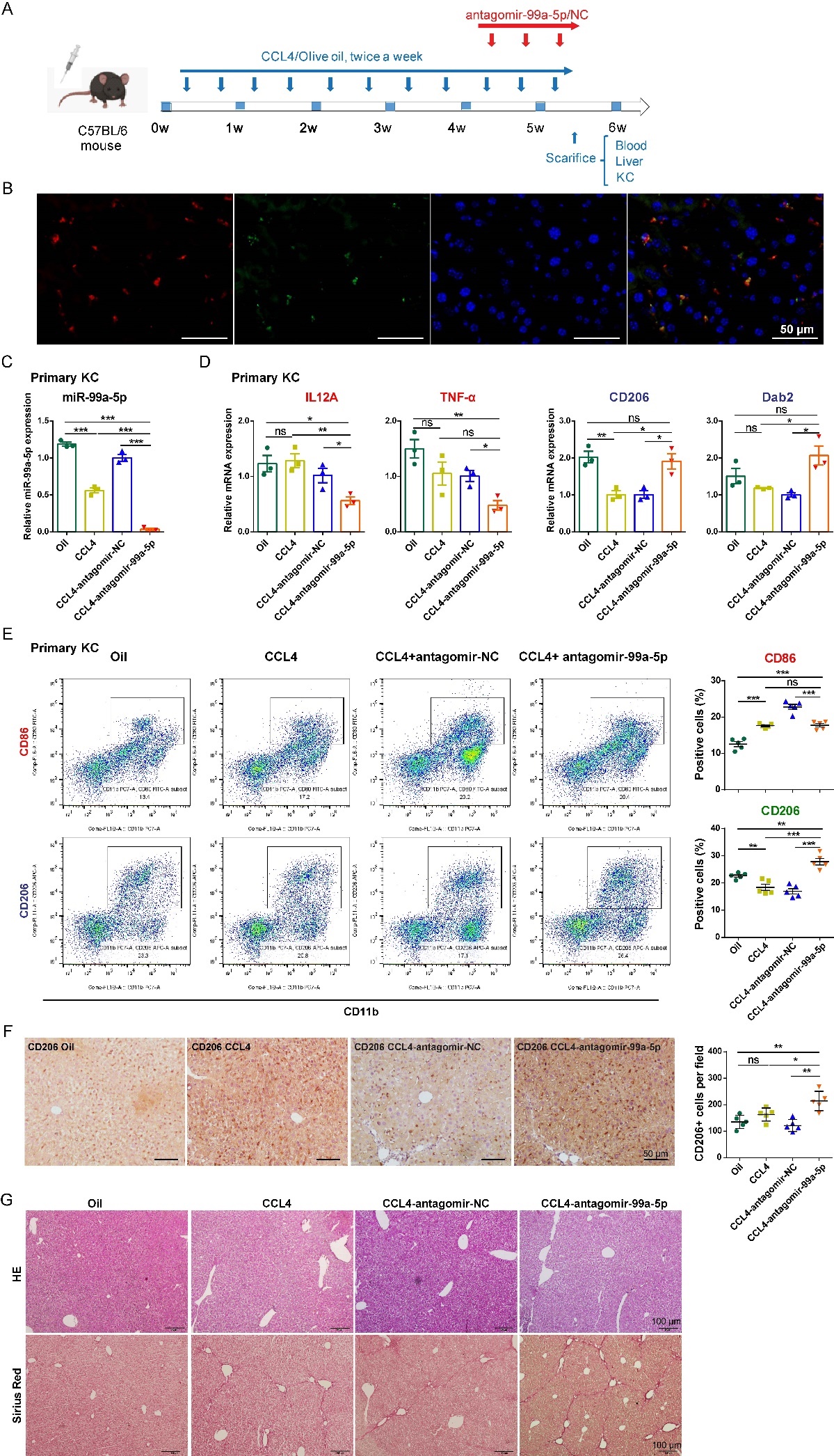


**Figure S10. The effects of** **antagomir-99a-5p on liver macrophage (KC) differentiation in a chronic liver injury mouse model.** (A) Schematic representation of antagomir-99a-5p treatment in a CCL4-induced chronic liver injury mouse model. (B) Uptake of antagomir by KCs. KCs were labeled with immunofluorescence staining of CD68 (Cy3, red), miRNA antagomir negative control (antagomir-NC). was labeled with FAM (green), and nuclei were counterstained with DAPI (blue) in frozen sections of mouse livers. Scale bar = 50 μm. (C) The expression of miR-99a-5p in KCs was determined by qRT-PCR and normalized to that of U6snRNA and to the miRNA antagomir negative control (antagomir-NC). (D) The expression of mRNAs of 4 genes associated with HSC-sEV-induced macrophage differentiation in a chronic liver injury mouse model was determined by qRT-PCR. The relative gene expression was normalized to that of β-Actin and then to antagomir-NC. (E) Surface marker expression in liver macrophages was determined by flow cytometry. Representative flow cytometry images and statistical histograms for CD86- and CD206-positive cells are provided. (F) Immunohistochemical staining of CD206 in liver tissue sections from each group. Representative images are provided, and positively stained cells are dark brown, scale bar = 50 μm. (G) H&E and Sirius Red staining of liver sections from each group. Representative images from at least five mice for each group are provided. Scale bar = 100 μm. Data from at least five mice for each group are presented as the mean ± SEM, and statistical significance was determined by one-way ANOVA, *** *p* < 0.001, ** *p* < 0.01, * *p* < 0.05, ns, not significant.


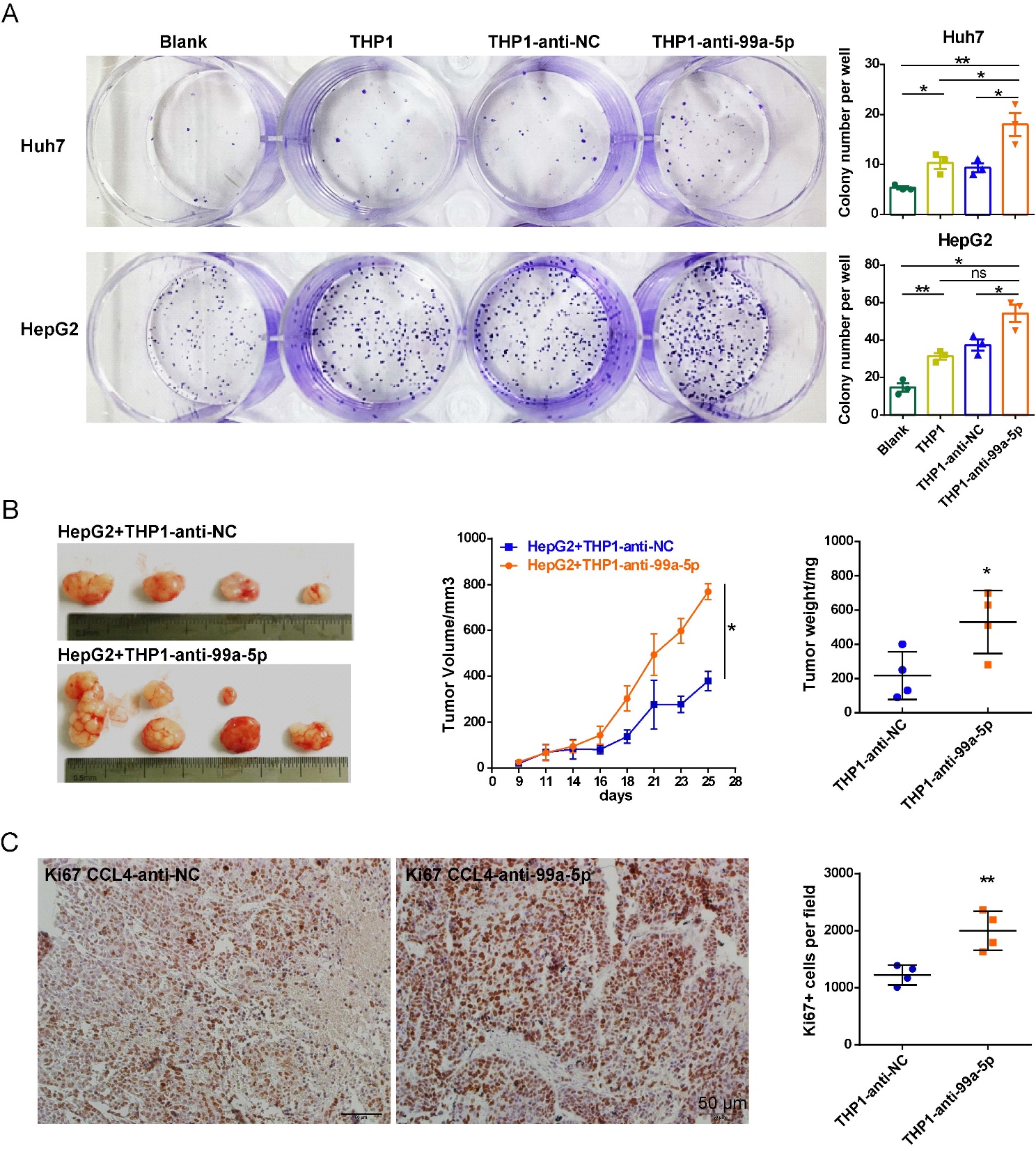


**Figure S11. Anti-miR-99a-5p-educated macrophages promoted the growth of hepatocellular carcinoma.** THP-1 macrophages were pretreated with anti-miR-99a-5p or anti-miR negative control (anti-NC) for 8 hr. (A) Colony formation of Huh7 or HepG2 cells cocultured with anti-miR-99a-5p-pretreated THP-1 macrophages at a ratio of 5:1. (B) Nude mouse xenograft experiments. HepG2 cells were mixed and subcutaneously cotransplanted with anti-miR-99a-5p-pretreated THP-1 macrophages. The size of the tumor was measured at the indicated time points. The weight of the tumor was obtained on Day25. (C) Immunohistochemical staining of Ki67 in paraffin sections of xenografts, scale bar = 50 μm. The experiments performed with THP-1 macrophages pretreated with anti-NC served as a negative control. Representative images are provided. Data from at least three wells (A) or five mice (B, C) for each group are shown as the mean ± SEM. Statistical significance was determined by one-way ANOVA, ** *p* < 0.01, * *p* < 0.05, ns, not significant.


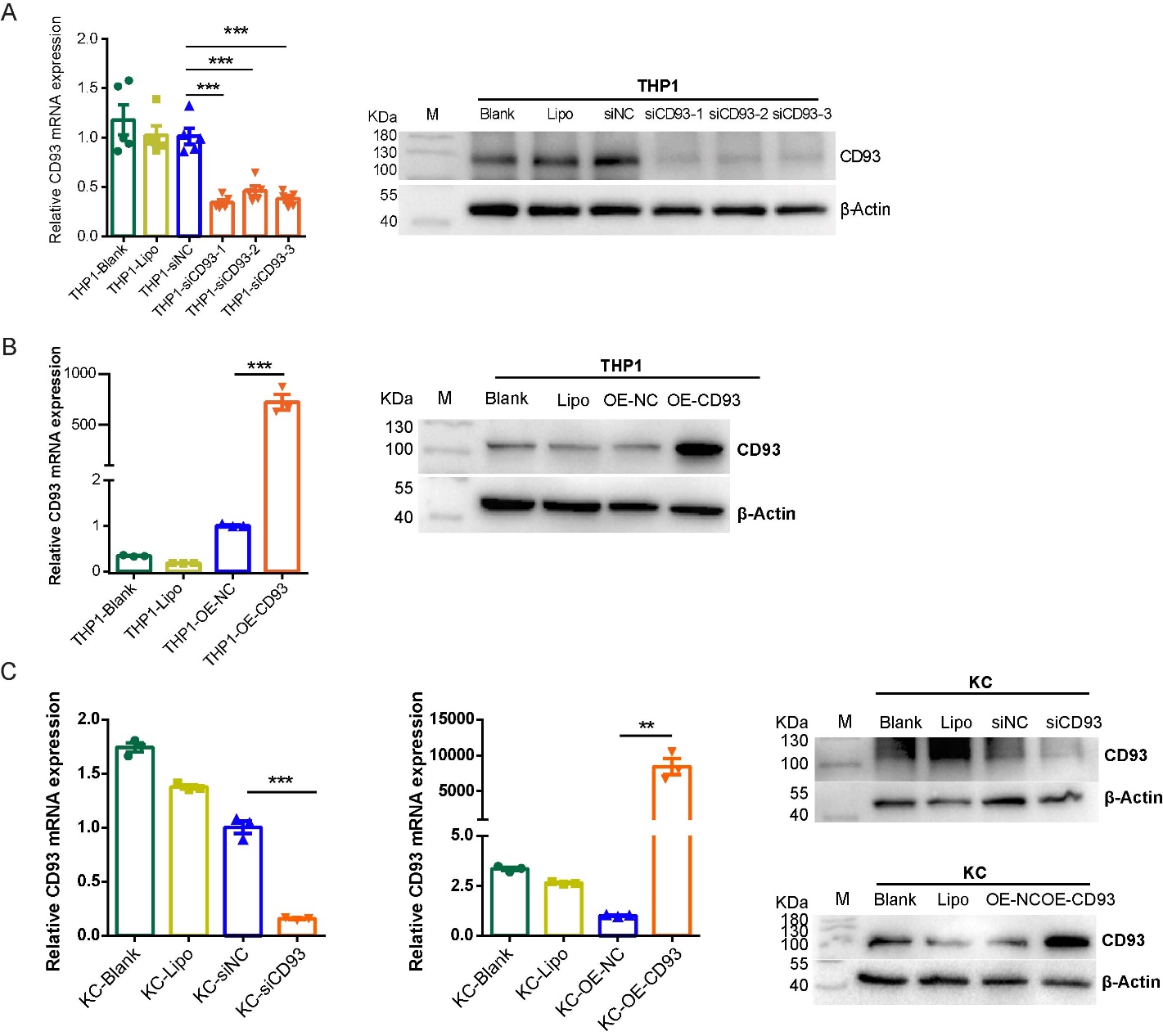


**Figure S12.** **The effects of CD93 on macrophage differentiation.** (A) CD93 siRNAs reduced the expression of CD93 in human THP-1 macrophages as detected by qRT-PCR and western blotting. The siCD93-1 was used in the following experiments. (B) CD93 overexpression plasmid (OE-CD93) increased the expression of CD93 in THP1 cells as detected by qRT-PCR and western blotting. (C) CD93 siRNAs reduced the expression of CD93 in rat primary KCs as detected by qRT-PCR and western blotting; OE-CD93 increased the expression of CD93 in in rat primary KCs as detected by qRT-PCR and western blotting. The relative gene expression was normalized to β-Actin in qRT-PCR experiments, and then to negative control (NC). Red font, genes highly expressed in 3dHSC-sEV cocultured KCs, green font, genes highly expressed in 14dHSC-sEV cocultured KCs. Data from at least three independent experiments are shown as the mean ± SEM. Statistical significance was determined by Student’s t test or one-way ANOVA, *** *p* < 0.001, ** *p* < 0.01.


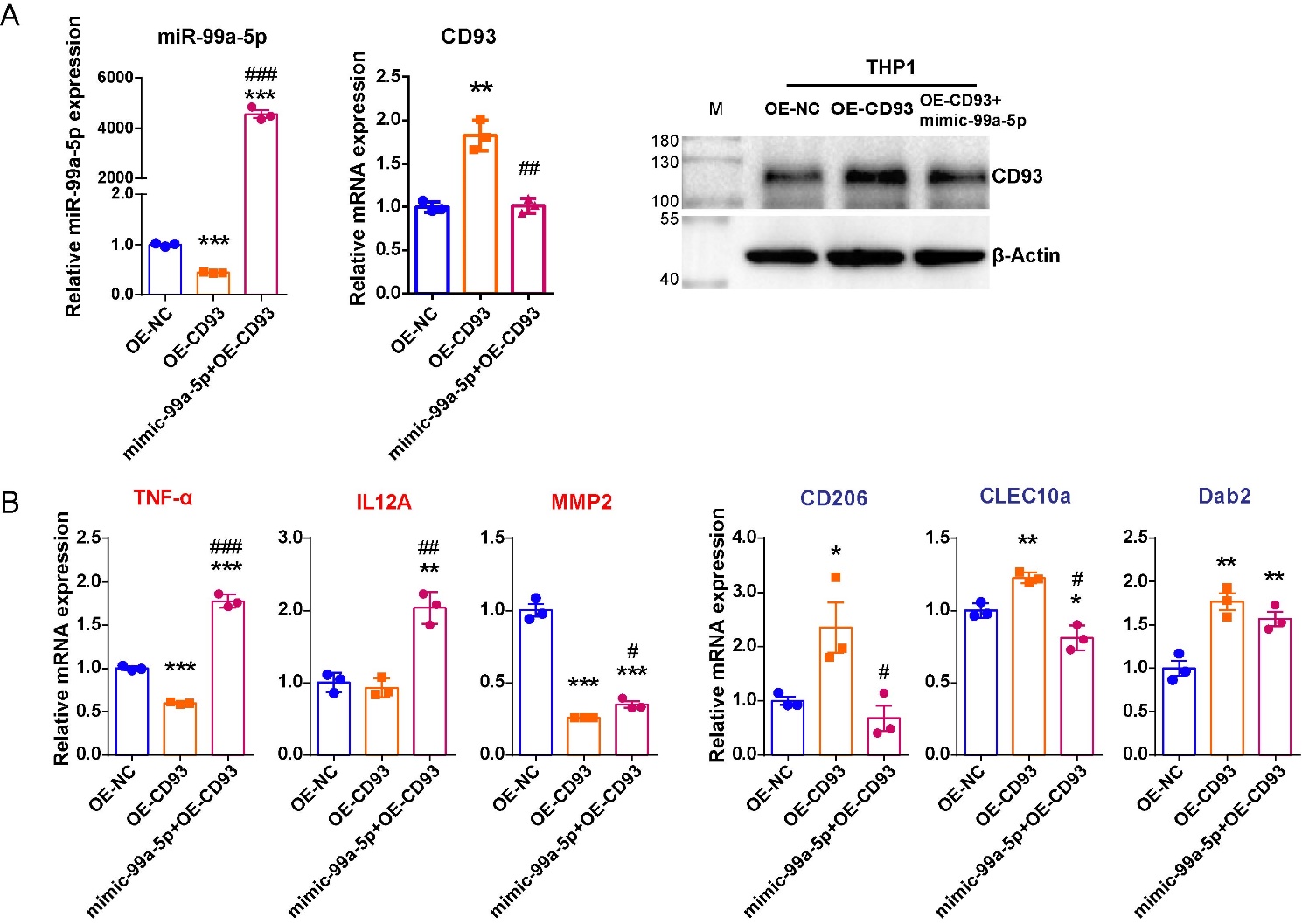


**Figure S13. Short-term activated HSC-sEV-specific miR-99a-5p might influence macrophage differentiation by targeting CD93.** (A) The effects of the miR-99a-5p mimic on miR-99a-5p and CD93 expression in human THP-1 macrophages and CD93 overexpression plasmid (OE-CD93) transfected human THP-1 macrophages were measured by qRT-PCR. (B) The effects of the miR-99a-5p mimic on the expression of genes associated with macrophage differentiation in CD93 overexpressing human THP-1 macrophages were measured by qRT-PCR. The relative miR-99a-5p expression was normalized to that of U6snRNA, and the relative gene expression was normalized to β-Actin in qRT-PCR experiments and then to negative control (NC). Statistical significance was determined by Student’s t test. Compared to NC, *** *p* < 0.001, ** *p* < 0.01, * *p* < 0.05; compared to OE-CD93, ### *p* < 0.001, ## *p* < 0.01, #*p* < 0.05. NC, negative control. Red font, genes highly expressed in 3dHSC-sEV cocultured KCs, green font, genes highly expressed in 14dHSC-sEV cocultured KCs.

**Supplemental Tables**

**Table S1 Antibodies information**

| Antibodies | SOURCE | IDENTIFIER |
| --- | --- | --- |
| α-SMA | Abcam | Cat# ab124964, RRID:AB_11129103 |
| Collagen I | Abcam | Cat# ab34710, RRID:AB_731684 |
| Collagen I | Proteintech | Cat# 14695-1-AP, RRID:AB_2082037 |
| Desmin | Abcam | Cat# ab32362, RRID:AB_731901 |
| NF-κBp65 | Cell Signaling Technology | Cat# 6956, RRID:AB_10828935 |
| TNF-α | Boster Biological Technology | Cat# BA0131, RRID:AB_3076605 |
| IL-6 | Cell Signaling Technology | Cat# 12912, RRID:AB_2798059 |
| Ki67 | Abcam | Cat# ab16667, RRID:AB_302459 |
| CD68 | Boster Biological Technology | Cat# BA3638, RRID:AB_2813855 |
| CD31 | Abcam | Cat# ab222783, RRID:AB_2905525 |
| CD206 (MRC1) | Boster Biological Technology | Cat# A02285-2, RRID:AB_3081542 |
| iNOS | Boster Biological Technology | Cat# BA0362, RRID: AB_3251498 |
| CD93 | Abcam | Cat# ab134079, RRID:AB_2909416 |
| CD9 | System Biosciences | Cat# EXOAB-CD9A-1, RRID:AB_2687469 |
| CD63 | System Biosciences | Cat# EXOAB-CD63A-1, RRID:AB_2561274 |
| CD81 | System Biosciences | Cat# EXOAB-CD81A-1, RRID:AB_2819191 |
| β-actin | Cell Signaling Technology | Cat# 4970, RRID:AB_2223172 |
| GAPDH | Beyotime | Cat# AG019, RRID:AB_2861160 |
| CD14-FITC | BioLegend | Clone: 63D3 Cat# 367115, RRID:AB_2571928 |
| CD86-PE/Cyanine5 | BioLegend | Clone: IT2.2 Cat# 305407, RRID:AB_314527 |
| CD206-APC | BioLegend | Clone: 15-2 Cat# 321109, RRID:AB_571884 |
| CD45-APC | Miltenyi Biotec | Clone: REA504 Cat# 130107790, RRID:AB_2658263 |
| CD31-PE | Miltenyi Biotec | Clone: REA396 Cat# 130116505, RRID:AB_2727579 |
| CD86-PE | BD Biosciences | Clone: 24F Cat# 551396, RRID:AB_394180 |
| F4/80-PE | BioLegend | Clone: BM8 Cat# 123109, RRID:AB_893498 |
| CD11b-PE/Cyanine7 | BioLegend | Clone: M1/70 Cat# 101215, RRID:AB_312798 |
| CD86-FITC | Thermo Fisher Scientific | Clone: GL1 Cat# 11086282, RRID:AB_465148 |
| CD206-APC | Thermo Fisher Scientific | Clone: MR6F3 Cat# 17206182, RRID:AB_2637420 |
| CD11b/c-PE | Miltenyi Biotec | Clone: REA325 Cat# 130105274, RRID:AB_2654683 |
| CD16/32 | BioLegend | Cat# 156603, RRID:AB_2783137 |

**Table S2. Primers used for quantitative reverse transcription polymerase chain reaction (qRT-PCR) for mRNA and miRNA quantification**

mRNA (Rattus norvegicus)

| **Gene** | **Sense prime (5'-3')** | **Antisense primer (5'-3')** |
| --- | --- | --- |
| β-Actin | ATGACGATATCGCTGCGCTC | CCATACCCACCATCACACCC |
| Traf1 | CACAGGGCTGGTCTCTACTT | GATTCCAGCCTCAGCCACAT |
| IL12a | GGCCAGGGTCATACCAGTCT | ACC ATG TCG TCC GTG GTCTT |
| Mmp2 | CACGATTGGTCTGGGGAGTG | CCTTGGGGCAGCCATAGAAA |
| CD86 | GCA AGG ATACCCGAAACCTAC | CCGGGA ATG GAA GAGATAGGC |
| CD206 (Mrc1) | ACTCTGGGCCATGAGACTCC | TGAACGGAGATGGCGCTTAG |
| CD74 | GACCCGTGAACTACCCACAG | TTGGCCCATATCCTGCTTGG |
| Adgre1 | AGCTGTCTTCCCGACTTTCG | CCTGCTTGGCACTGCTCTAT |
| Dab2 | GTGTGGCCACCTTTACCCTT | TCGAAATGGTCCTTGGGTGG |
| CD93 | CCATCTCACTCTTGCTGGCT | TTGTCTTCAGCAGTCCGTCC |

mRNA (Homo sapiens)

| **Gene** | **Sense prime (5'-3')** | **Antisense primer (5'-3')** |
| --- | --- | --- |
| β-Actin | TTGTTACAGGAAGTCCCTTGCC | ATGCTATCACCTCCCCTGTGTG |
| 18S | CAGCCACCCGAGATTGAGCA | TAGTAGCGACGGGCGGTGTG |
| α- SMA | CTTGGACTCTGTGCCCTCATC | TGGCTCTTTCATCGGGTTTAG |
| COLI | GTGCGAGACGTGATCTGTGA | TTGGTCGGTGGGTGACTCTG |
| TRAF1 | CAGTCCCAGGCAAGATAGGG | CCCTGGATGGTGACTGAAGG |
| IL12a | TCCAGAAGGCCAGACAAACTCTA | GCCAGGCAACTCCCATTAGTTA |
| MMP2 | GGACACATCTGGGCAGTTGCTA | GTCCAGATCAGGTGTGTAGCC |
| CD86 | GCACAGACACACGGATGAGT | GGGTCCAACTGTCCGAATCA |
| CD206 (MRC1) | ACCTGCGACAGTAAACGAGG | TGTCTCCGCTTCATGCCATT |
| CD74 | CACCAAGTATGGCAACATGACAGA | TCCAGTTCCAGTGACTCTTCCA |
| CLEC10a | CCAGGAACACACTACGCAGA | GTGGAGGCATTGTTGTTGAGAG |
| DAB2 | CGGGCATTTGGTTACGTGTG | CTGGTCAACACCCGATTTCAG |
| CD93 | TCTGATGGAAACCACTGGGC | CGAGAAGTTAGCAGGTGGCA |

mRNA (Mus musculus)

| **Gene** | **Sense prime (5'-3')** | **Antisense primer (5'-3')** |
| --- | --- | --- |
| β-Actin | GGCTGTATTCCCCTCCATCG | CCAGTTGGTAACGCCATGT |
| IL12a | ATGATGACCCTGTGCCTTGG | CACCCTGTTGATGGTCACGA |
| Tnf-α | CTGTAGCCCACGTCGTAG | TTGAGATCCATCCCGTTG |
| CD206 (Mrc1) | CTCTGTTCAGCTATTGGACGC | CGGAATTTCTGGGATTCAGCTTC |
| Dab2 | CAGTCCCGCAGTCGAACTT | GTAGACAACAGGCGTCCAGG |

miRNAs and U6 snRNA

| **Gene** | **Sense prime (5'-3')** | **Antisense primer (5'-3') *** | **Tm (℃)** |
| --- | --- | --- | --- |
| let-7f-5p | CGGTGAGGTAGTAGATTGTATAGTT |  | 54 |
| miR-10a-5p | TACCCTGTAGATCCGAATTTGTG |  | 58.8 |
| miR-125a-5p | TCCCTGAGACCCTTTAACCTGTGA |  | 57 |
| miR-125b-1-3p | ACGGGTTAGGCTCTTGGGAGCT |  | 59 |
| miR-127-3p | TCGGATCCGTCTGAGCTTGGCT |  | 59 |
| miR-139-5p | TCTACAGTGCACGTGTCTCCAG |  | 58.5 |
| miR-143-3p | TGAGATGAAGCACTGTAGCTCA |  | 53 |
| miR-146b-5p | TGAGAACTGAATTCCATAGGCTGT |  | 54 |
| miR-27a-3p | TTCACAGTGGCTAAGTTCCGC |  | 54 |
| miR-27b-3p | TTCACAGTGGCTAAGTTCTGC |  | 52 |
| miR-30a-5p | TGTAAACATCCTCGACTGGAAG |  | 57 |
| miR-30b-5p | TGTAAACATCCTACACTCAGCT |  | 52 |
| miR-423-5p | TGAGGGGCAGAGAGCGAGACTTTT |  | 59 |
| miR-486-5p | TCCTGTACTGAGCTGCCCCGA |  | 58 |
| miR-99a-5p | AACCCGTAGATCCGATCTTGTG |  | 55 |
| miR-221-3p | AGCTACATTGTCTGCTGGGTTTC |  | 60.4 |
| U6 | CTCGCTTCGGCAGCACA | AACGCTTCACGAATTTGCGT |  |

Note: ***** Sense primers for mature miRNAs were provided here, antisense primer was provided by RiboBio as Universal q-PCR Primer.

**Table S3. Canonical pathway analysis of the differentially expressed genes between KCs and MOs.**

| **Ingenuity Canonical Pathways** | **-log(p-value)** | **z-score** | **Ingenuity Canonical Pathways** | **-log(p-value)** | **z-score** |
| --- | --- | --- | --- | --- | --- |
| Synaptogenesis Signaling Pathway | 3.08 | 5.692 | **LPS/IL-1 Mediated Inhibition of RXR Function** | 3.50 | 2.714 |
| IL-15 Production | 5.44 | 5.099 | Adrenomedullin signaling pathway | 1.76 | 2.711 |
| CREB Signaling in Neurons | 9.85 | 5.027 | D-myo-inositol-5-phosphate Metabolism | 1.80 | 2.683 |
| **Hepatic Fibrosis Signaling Pathway** | 13.90 | 4.866 | Calcium-induced T Lymphocyte Apoptosis | 4.84 | 2.668 |
| NF-κB Signaling | 2.89 | 4.707 | Cdc42 Signaling | 1.55 | 2.646 |
| **IL-17 Signaling** | 3.26 | 4.642 | Acute Phase Response Signaling | 5.98 | 2.600 |
| **HER-2 Signaling in Breast Cancer** | 1.60 | 4.491 | Factors Promoting Cardiogenesis in Vertebrates | 3.75 | 2.600 |
| Breast Cancer Regulation by Stathmin1 | 11.60 | 4.445 | Endothelin-1 Signaling | 2.58 | 2.600 |
| Cardiac Hypertrophy Signaling (Enhanced) | 7.23 | 4.431 | Sperm Motility | 7.09 | 2.524 |
| Dendritic Cell Maturation | 3.73 | 4.426 | Cholecystokinin/Gastrin-mediated Signaling | 3.25 | 2.524 |
| Integrin Signaling | 3.96 | 4.271 | MSP-RON Signaling In Cancer Cells Pathway | 1.92 | 2.524 |
| **Regulation Of The Epithelial Mesenchymal Transition By Growth Factors Pathway** | 2.28 | 4.082 | eNOS Signaling | 1.74 | 2.524 |
| Cell Cycle Control of Chromosomal Replication | 5.22 | 4.000 | CXCR4 Signaling | 1.53 | 2.524 |
| iCOS-iCOSL Signaling in T Helper Cells | 2.09 | 4.000 | **PDGF Signaling** | 2.04 | 2.496 |
| STAT3 Pathway | 12.60 | 3.922 | Mouse Embryonic Stem Cell Pluripotency | 1.41 | 2.496 |
| Phospholipase C Signaling | 3.34 | 3.900 | Cell Cycle Regulation by BTG Family Proteins | 2.64 | 2.449 |
| Systemic Lupus Erythematosus In B Cell Signaling Pathway | 2.27 | 3.888 | Differential Regulation of Cytokine Production in Intestinal Epithelial Cells by IL-17A and IL-17F | 2.07 | 2.449 |
| Role of Hypercytokinemia/hyperchemokinemia in the Pathogenesis of Influenza | 2.43 | 3.873 | ERK/MAPK Signaling | 1.89 | 2.400 |
| IL-8 Signaling | 4.52 | 3.772 | Aryl Hydrocarbon Receptor Signaling | 5.45 | 2.357 |
| Kinetochore Metaphase Signaling Pathway | 14.20 | 3.651 | Osteoarthritis Pathway | 6.82 | 2.335 |
| **Tumor Microenvironment Pathway** | 4.09 | 3.651 | Estrogen-mediated S-phase Entry | 3.88 | 2.333 |
| p38 MAPK Signaling | 2.52 | 3.638 | Chondroitin Sulfate Biosynthesis (Late Stages) | 1.85 | 2.333 |
| Neuroinflammation Signaling Pathway | 3.72 | 3.569 | Chondroitin Sulfate Biosynthesis | 1.45 | 2.333 |
| Colorectal Cancer Metastasis Signaling | 4.11 | 3.550 | Toll-like Receptor Signaling | 1.37 | 2.333 |
| Signaling by Rho Family GTPases | 2.33 | 3.530 | Dermatan Sulfate Biosynthesis | 1.33 | 2.333 |
| GP6 Signaling Pathway | 7.87 | 3.528 | PAK Signaling | 1.32 | 2.333 |
| Thrombin Signaling | 3.14 | 3.528 | Chemokine Signaling | 1.92 | 2.309 |
| Reelin Signaling in Neurons | 2.02 | 3.500 | ILK Signaling | 5.01 | 2.191 |
| Tec Kinase Signaling | 2.46 | 3.441 | Apelin Endothelial Signaling Pathway | 2.65 | 2.183 |
| Crosstalk between Dendritic Cells and Natural Killer Cells | 2.29 | 3.317 | G Beta Gamma Signaling | 2.02 | 2.183 |
| CD28 Signaling in T Helper Cells | 1.74 | 3.317 | **Th2 Pathway** | 3.63 | 2.138 |
| HMGB1 Signaling | 3.09 | 3.300 | Corticotropin Releasing Hormone Signaling | 3.59 | 2.132 |
| **IL-6 Signaling** | **2.20** | **3.300** | Inhibition of Angiogenesis by TSP1 | 3.59 | 2.121 |
| HIF1α Signaling | 2.93 | 3.286 | Apelin Liver Signaling Pathway | 3.12 | 2.121 |
| Estrogen Receptor Signaling | 1.40 | 3.212 | Macropinocytosis Signaling | 2.11 | 2.121 |
| Role of Pattern Recognition Receptors in Recognition of Bacteria and Viruses | 4.36 | 3.207 | CCR5 Signaling in Macrophages | 1.72 | 2.121 |
| Glioma Signaling | 3.29 | 3.207 | UVC-Induced MAPK Signaling | 1.69 | 2.121 |
| Neuregulin Signaling | 3.13 | 3.207 | Ovarian Cancer Signaling | 3.89 | 2.111 |
| TREM1 Signaling | 2.59 | 3.207 | Acute Myeloid Leukemia Signaling | 1.57 | 2.111 |
| ErbB Signaling | 2.07 | 3.207 | Gαq Signaling | 1.53 | 2.065 |
| Pancreatic Adenocarcinoma Signaling | 3.79 | 3.051 | Prostanoid Biosynthesis | 3.20 | 2.000 |
| **Fcγ Receptor-mediated Phagocytosis in Macrophages and Monocytes** | 1.40 | 3.051 | NAD biosynthesis II (from tryptophan) | 1.90 | 2.000 |
| VEGF Family Ligand-Receptor Interactions | 3.94 | 3.000 | Role of NANOG in Mammalian Embryonic Stem Cell Pluripotency | 1.51 | 2.000 |
| Ephrin Receptor Signaling | 3.52 | 3.000 | Tryptophan Degradation III (Eukaryotic) | 1.46 | 2.000 |
| Paxillin Signaling | 2.98 | 2.887 | **Endocannabinoid Cancer Inhibition Pathway** | **1.65** | **-2.065** |
| Glioblastoma Multiforme Signaling | 4.63 | 2.858 | HIPPO signaling | 1.72 | -2.530 |
| GNRH Signaling | 1.38 | 2.840 | **PD-1, PD-L1 cancer immunotherapy pathway** | **1.32** | **-3.051** |
| Renin-Angiotensin Signaling | 2.90 | 2.828 | **PTEN Signaling** | **2.17** | **-3.357** |
| Leukocyte Extravasation Signaling | 4.08 | 2.785 | RhoGDI Signaling | 2.85 | -3.545 |
| Natural Killer Cell Signaling | 2.29 | 2.746 | **PPAR Signaling** | **1.35** | **-3.606** |
| Note: Only the pathways which had a -log(p-value) > 1.3 (*p* < 0.05), and z-score > 2.0 or < -2.0 were included. | | | | | |

**Table S4. Differentially expressed genes between 3dHSC and 14dHSC-sEVs co-cultured KCs**

Please refer to online Supplemental Spreadsheets.

**Table S5. Small RNA annotation**

Please refer to online Supplemental Spreadsheets.

**Table S6. The miRNAs detected in primary rat HSCs and HSC-sEVs**

Please refer to online Supplemental Spreadsheets.

**Table S7. Differentially expressed miRNAs in 3dHSCs vs 14dHSCs**

| **miRNA_id** | **BaseMean** | **BaseMean_3dHSCs** | **BaseMean_14dHSCs** | **Fold-Change** | **Log2 Fold-Chang** | **P-val** | **padj** | **Length** |
| --- | --- | --- | --- | --- | --- | --- | --- | --- |
| rno-miR-146b-5p | 294942.16 | 393337.62 | 196546.71 | **2.001** | **1.001** | 0.056 | 1.000 | 24 |
| rno-miR-30d-5p | 253817.27 | 338902.39 | 168732.14 | **2.009** | **1.006** | 0.086 | 1.000 | 22 |
| rno-miR-99a-5p | 210648.65 | 296217.44 | 125079.86 | **2.368** | **1.244** | 0.033 | 1.000 | 22 |
| rno-miR-27b-3p | 95334.56 | 47958.16 | 142710.95 | **0.336** | **-1.573** | 0.011 | 0.558 | 21 |
| rno-miR-199a-3p | 90785.88 | 1496.60 | 180075.17 | **0.008** | **-6.911** | 0.088 | 1.000 | 22 |
| rno-miR-221-3p | 83273.71 | 54706.82 | 111840.59 | **0.489** | **-1.032** | 0.104 | 1.000 | 23 |
| rno-miR-126a-3p | 81330.45 | 155808.13 | 6852.77 | **22.737** | **4.507** | 0.002 | 0.192 | 22 |
| rno-miR-143-3p | 56965.09 | 3583.37 | 110346.80 | **0.032** | **-4.945** | 0.022 | 0.781 | 22 |
| rno-miR-199a-5p | 30335.83 | 274.51 | 60397.15 | **0.005** | **-7.781** | 0.061 | 1.000 | 23 |
| rno-miR-122-5p | 22393.52 | 38611.48 | 6175.55 | **6.252** | **2.644** | 0.001 | 0.088 | 22 |
| rno-miR-152-3p | 15684.73 | 1790.27 | 29579.19 | **0.061** | **-4.046** | 0.022 | 0.785 | 21 |
| rno-miR-34c-5p | 11907.98 | 1997.55 | 21818.41 | **0.092** | **-3.449** | 0.000 | 0.058 | 23 |
| rno-miR-125b-1-3p | 3361.17 | 254.77 | 6467.58 | **0.039** | **-4.666** | 0.086 | 1.000 | 22 |
| rno-miR-218a-5p | 3256.96 | 858.96 | 5654.96 | **0.152** | **-2.719** | 0.002 | 0.198 | 21 |
| rno-miR-150-5p | 2731.79 | 5074.93 | 388.64 | **13.058** | **3.707** | 0.000 | 0.027 | 22 |
| rno-miR-23b-3p | 2636.48 | 1256.64 | 4016.31 | **0.313** | **-1.676** | 0.054 | 1.000 | 21 |
| rno-miR-330-5p | 2420.85 | 1141.82 | 3699.88 | **0.309** | **-1.696** | 0.050 | 1.000 | 22 |
| novel784_mature | 1946.66 | 3255.27 | 638.05 | **5.102** | **2.351** | 0.014 | 0.604 | 20 |
| rno-miR-139-5p | 1565.17 | 2952.17 | 178.17 | **16.569** | **4.050** | 0.000 | 0.027 | 22 |
| rno-miR-145-5p | 1455.59 | 109.23 | 2801.94 | **0.039** | **-4.681** | 0.076 | 1.000 | 23 |
| rno-miR-132-3p | 1429.83 | 333.88 | 2525.79 | **0.132** | **-2.919** | 0.017 | 0.682 | 22 |
| rno-miR-107-3p | 1388.04 | 699.95 | 2076.13 | **0.337** | **-1.569** | 0.083 | 1.000 | 23 |
| rno-miR-31a-5p | 1232.92 | 46.36 | 2419.49 | **0.019** | **-5.706** | 0.082 | 1.000 | 22 |
| rno-miR-34b-3p | 1070.47 | 220.68 | 1920.26 | **0.115** | **-3.121** | 0.002 | 0.192 | 22 |
| rno-miR-224-5p | 1012.34 | 17.94 | 2006.75 | **0.009** | **-6.806** | 0.081 | 1.000 | 22 |
| rno-miR-132-5p | 963.67 | 216.11 | 1711.22 | **0.126** | **-2.985** | 0.032 | 1.000 | 22 |
| rno-miR-184 | 955.01 | 295.20 | 1614.81 | **0.183** | **-2.452** | 0.014 | 0.604 | 22 |
| rno-miR-212-5p | 799.09 | 104.42 | 1493.77 | **0.070** | **-3.838** | 0.003 | 0.254 | 24 |
| rno-miR-342-3p | 787.79 | 1258.42 | 317.15 | **3.968** | **1.988** | 0.045 | 1.000 | 23 |
| rno-miR-126a-5p | 634.86 | 1189.59 | 80.13 | **14.846** | **3.892** | 0.000 | 0.058 | 21 |
| rno-miR-152-5p | 616.05 | 82.25 | 1149.86 | **0.072** | **-3.805** | 0.072 | 1.000 | 23 |
| rno-miR-214-3p | 472.53 | 3.51 | 941.54 | **0.004** | **-8.066** | 0.048 | 1.000 | 21 |
| novel906_mature | 416.32 | 32.85 | 799.79 | **0.041** | **-4.606** | 0.000 | 0.027 | 18 |
| rno-miR-149-5p | 349.75 | 18.83 | 680.67 | **0.028** | **-5.176** | 0.023 | 0.797 | 23 |
| rno-miR-200a-3p | 294.01 | 557.09 | 30.93 | **18.010** | **4.171** | 0.001 | 0.077 | 22 |
| rno-miR-130a-3p | 264.45 | 77.74 | 451.16 | **0.172** | **-2.537** | 0.025 | 0.806 | 22 |
| rno-miR-34c-3p | 233.35 | 74.25 | 392.44 | **0.189** | **-2.402** | 0.036 | 1.000 | 22 |
| novel438_mature | 210.43 | 85.18 | 335.68 | **0.254** | **-1.978** | 0.078 | 1.000 | 22 |
| rno-miR-212-3p | 203.70 | 37.05 | 370.34 | **0.100** | **-3.321** | 0.011 | 0.558 | 22 |
| rno-miR-129-5p | 187.09 | 28.42 | 345.75 | **0.082** | **-3.605** | 0.085 | 1.000 | 21 |
| rno-miR-200b-3p | 163.73 | 303.43 | 24.02 | **12.630** | **3.659** | 0.003 | 0.254 | 23 |
| novel922_mature | 146.47 | 43.38 | 249.56 | **0.174** | **-2.524** | 0.041 | 1.000 | 19 |
| novel18_star | 145.56 | 4.56 | 286.56 | **0.016** | **-5.974** | 0.000 | 0.013 | 22 |
| rno-miR-31a-3p | 141.38 | 3.93 | 278.83 | **0.014** | **-6.147** | 0.087 | 1.000 | 23 |
| rno-miR-196c-5p | 117.34 | 0.00 | 234.69 | **0.000** | **#NUM!** | 0.000 | 0.000 | 22 |
| novel452_star | 115.22 | 10.44 | 220.01 | **0.047** | **-4.398** | 0.006 | 0.359 | 23 |
| rno-miR-143-5p | 103.38 | 3.51 | 203.25 | **0.017** | **-5.854** | 0.024 | 0.806 | 21 |
| rno-miR-582-3p | 103.09 | 31.36 | 174.81 | **0.179** | **-2.479** | 0.044 | 1.000 | 22 |
| Note: Only those miRNAs that had a basemean>100, and the differential expression showed a *p* < 0.10 were included. | | | | | | | | |

**Table S8. Differentially transported miRNAs by 3dHSC-sEVs vs 14dHSC-sEVs**

| **miRNA_id** | **BaseMean** | **BaseMean_3dHSC-sEVs** | | **BaseMean_14dHSC-sEVs** | **Fold-Change** | **Log2Fold-Chang** | **P-val** | **padj** | **Length** |
| --- | --- | --- | --- | --- | --- | --- | --- | --- | --- |
| rno-miR-423-5p | 224186.47 | 93590.60 | 354782.33 | | **0.264** | **-1.922** | 0.095 | 0.830 | 24 |
| rno-miR-10a-5p | 87252.72 | 156786.72 | 17718.73 | | **8.849** | **3.145** | 0.000 | 0.025 | 23 |
| rno-miR-146a-5p | 76251.94 | 44057.43 | 108446.45 | | **0.406** | **-1.300** | 0.090 | 0.829 | 22 |
| rno-let-7b-5p | 31151.16 | 46865.46 | 15436.86 | | **3.036** | **1.602** | 0.029 | 0.493 | 22 |
| rno-miR-99a-5p | 28740.53 | 50205.02 | 7276.04 | | **6.900** | **2.787** | 0.000 | 0.051 | 22 |
| novel784_mature | 15381.73 | 23631.05 | 7132.42 | | **3.313** | **1.728** | 0.030 | 0.493 | 20 |
| rno-let-7f-5p | 12745.07 | 20004.56 | 5485.59 | | **3.647** | **1.867** | 0.017 | 0.404 | 22 |
| rno-miR-30a-5p | 10819.29 | 15743.59 | 5895.00 | | **2.671** | **1.417** | 0.072 | 0.737 | 22 |
| rno-miR-146b-5p | 6078.41 | 1339.35 | 10817.47 | | **0.124** | **-3.014** | 0.001 | 0.079 | 24 |
| rno-miR-101a-3p | 5588.01 | 9646.51 | 1529.51 | | **6.307** | **2.657** | 0.068 | 0.719 | 21 |
| rno-let-7a-5p | 4335.51 | 6868.93 | 1802.09 | | **3.812** | **1.930** | 0.023 | 0.460 | 22 |
| novel329_mature | 4160.42 | 1693.32 | 6627.51 | | **0.255** | **-1.969** | 0.026 | 0.463 | 19 |
| rno-let-7c-5p | 3838.99 | 6129.29 | 1548.68 | | **3.958** | **1.985** | 0.025 | 0.463 | 22 |
| rno-miR-100-5p | 3417.81 | 5199.62 | 1636.00 | | **3.178** | **1.668** | 0.042 | 0.574 | 22 |
| rno-miR-221-3p | 1978.42 | 276.71 | 3680.13 | | **0.075** | **-3.733** | 0.000 | 0.025 | 23 |
| rno-miR-30e-3p | 1849.21 | 3557.56 | 140.87 | | **25.255** | **4.658** | 0.000 | 0.009 | 22 |
| rno-miR-1b | 1350.88 | 694.50 | 2007.27 | | **0.346** | **-1.531** | 0.098 | 0.830 | 22 |
| rno-miR-126a-3p | 1322.47 | 2363.74 | 281.20 | | **8.406** | **3.071** | 0.027 | 0.479 | 22 |
| novel275_mature | 943.78 | 1512.82 | 374.74 | | **4.037** | **2.013** | 0.065 | 0.704 | 25 |
| novel671_mature | 834.77 | 403.69 | 1265.85 | | **0.319** | **-1.649** | 0.104 | 0.849 | 21 |
| rno-miR-192-5p | 827.90 | 1308.32 | 347.49 | | **3.765** | **1.913** | 0.058 | 0.686 | 21 |
| novel787_mature | 801.63 | 131.41 | 1471.86 | | **0.089** | **-3.485** | 0.002 | 0.151 | 24 |
| rno-miR-139-5p | 691.48 | 1324.47 | 58.48 | | **22.646** | **4.501** | 0.000 | 0.025 | 22 |
| rno-miR-760-3p | 592.17 | 129.11 | 1055.24 | | **0.122** | **-3.031** | 0.009 | 0.313 | 20 |
| rno-miR-101b-3p | 490.77 | 823.71 | 157.84 | | **5.219** | **2.384** | 0.035 | 0.523 | 21 |
| rno-miR-142-5p | 463.27 | 782.10 | 144.44 | | **5.415** | **2.437** | 0.035 | 0.523 | 21 |
| rno-miR-30c-5p | 456.82 | 711.37 | 202.28 | | **3.517** | **1.814** | 0.097 | 0.830 | 23 |
| novel728_mature | 429.57 | 31.40 | 827.75 | | **0.038** | **-4.720** | 0.000 | 0.045 | 24 |
| novel631_mature | 419.96 | 158.76 | 681.16 | | **0.233** | **-2.101** | 0.083 | 0.788 | 18 |
| rno-miR-30e-5p | 384.83 | 631.80 | 137.86 | | **4.583** | **2.196** | 0.074 | 0.743 | 22 |
| rno-miR-151-5p | 301.93 | 521.05 | 82.80 | | **6.293** | **2.654** | 0.031 | 0.493 | 21 |
| rno-miR-184 | 282.79 | 49.36 | 516.22 | | **0.096** | **-3.387** | 0.012 | 0.325 | 22 |
| novel386_mature | 239.38 | 460.32 | 18.44 | | **24.967** | **4.642** | 0.002 | 0.133 | 18 |
| novel375_mature | 239.06 | 447.79 | 30.33 | | **14.766** | **3.884** | 0.007 | 0.302 | 18 |
| novel525_mature | 235.06 | 8.87 | 461.24 | | **0.019** | **-5.700** | 0.100 | 0.845 | 18 |
| rno-miR-34c-5p | 200.97 | 54.23 | 347.71 | | **0.156** | **-2.681** | 0.045 | 0.589 | 23 |
| novel809_mature | 193.23 | 360.30 | 26.17 | | **13.769** | **3.783** | 0.009 | 0.313 | 18 |
| rno-miR-222-3p | 164.57 | 23.62 | 305.51 | | **0.077** | **-3.693** | 0.028 | 0.492 | 21 |
| rno-miR-335 | 162.63 | 318.63 | 6.62 | | **48.142** | **5.589** | 0.013 | 0.341 | 23 |
| novel324_mature | 156.35 | 312.70 | 0.00 | | **-** | **-** | 0.047 | 0.602 | 18 |
| rno-miR-466c-5p | 149.94 | 45.99 | 253.88 | | **0.181** | **-2.465** | 0.080 | 0.774 | 21 |
| rno-miR-125b-1-3p | 148.77 | 21.24 | 276.30 | | **0.077** | **-3.701** | 0.069 | 0.719 | 22 |
| rno-miR-511-3p | 141.57 | 272.73 | 10.40 | | **26.223** | **4.713** | 0.005 | 0.280 | 21 |
| rno-miR-322-3p | 138.74 | 237.30 | 40.18 | | **5.906** | **2.562** | 0.066 | 0.704 | 20 |
| rno-miR-212-5p | 135.27 | 7.98 | 262.57 | | **0.030** | **-5.041** | 0.003 | 0.187 | 24 |
| novel906_mature | 133.63 | 31.42 | 235.84 | | **0.133** | **-2.908** | 0.091 | 0.829 | 18 |
| novel286_mature | 131.03 | 38.69 | 223.36 | | **0.173** | **-2.529** | 0.091 | 0.829 | 20 |
| rno-miR-139-3p | 126.67 | 244.35 | 8.98 | | **27.203** | **4.766** | 0.006 | 0.291 | 22 |
| novel373_mature | 120.54 | 226.30 | 14.78 | | **15.314** | **3.937** | 0.019 | 0.423 | 18 |
| novel332_mature | 117.54 | 0.50 | 234.58 | | **0.002** | **-8.867** | 0.001 | 0.058 | 19 |
| rno-miR-342-3p | 115.55 | 214.55 | 16.55 | | **12.966** | **3.697** | 0.030 | 0.493 | 23 |
| rno-miR-10a-3p | 103.86 | 207.24 | 0.47 | | **438.376** | **8.776** | 0.000 | 0.025 | 22 |
| Note: Only those miRNAs that had a basemean >100, and the differential expression showed a *p* < 0.10 were included. | | | | | | | | |  |

**Table S9. The top 20 miRNAs with the highest abundance in primary rat HSC-sEVs**

| **miRNA_id** | **BaseMean_3dHSC_sEV** | **% of Total** | **BaseMean_14dHSC_sEV** | **% of Total** |
| --- | --- | --- | --- | --- |
| **rno-miR-122-5p** | 308293.19 | 24.74 | 149080.52 | 11.77 |
| **rno-miR-10a-5p** | 156786.72 | 12.58 | 17718.73 | 1.40 |
| **rno-miR-423-5p** | 93590.60 | 7.51 | 354782.33 | 28.00 |
| **rno-miR-22-3p** | 78780.35 | 6.32 | 150644.94 | 11.89 |
| **rno-miR-99a-5p** | 50205.02 | **4.03** | 7276.04 | 0.57 |
| **rno-miR-30d-5p** | 47159.37 | 3.78 | 27890.26 | 2.20 |
| **rno-let-7b-5p** | 46865.46 | 3.76 | 15436.86 | 1.22 |
| **rno-miR-151-3p** | 44904.27 | 3.60 | 49327.48 | 3.89 |
| **rno-miR-146a-5p** | 44057.43 | 3.54 | 108446.45 | 8.56 |
| **novel784_mature** | 23631.05 | 1.90 | 7132.42 | 0.56 |
| **rno-miR-29a-3p** | 23405.77 | 1.88 | 25103.51 | 1.98 |
| **rno-miR-21-5p** | 21362.67 | 1.71 | 16060.18 | 1.27 |
| **rno-let-7f-5p** | 20004.56 | 1.61 | 5485.59 | 0.43 |
| **rno-miR-99b-5p** | 17054.33 | 1.37 | 35487.04 | 2.80 |
| **rno-miR-148a-3p** | 15849.60 | 1.27 | 10490.73 | 0.83 |
| **rno-miR-3557-5p** | 15498.95 | 1.24 | 35468.67 | 2.80 |
| **rno-miR-320-3p** | 11449.12 | 0.92 | 12512.13 | 0.99 |
| **rno-miR-378a-3p** | 10481.37 | 0.84 | 15694.00 | 1.24 |
| **rno-miR-24-3p** | 9141.74 | 0.73 | 18342.47 | 1.45 |
| **rno-let-7i-5p** | 7402.29 | 0.59 | 15888.34 | 1.25 |
| **Total** |  | **83.94** |  | **85.10** |
